# Hemiacetal-less rapamycin derivatives designed and produced by genetic engineering of a type I polyketide synthase

**DOI:** 10.1038/s41598-021-88583-z

**Published:** 2021-05-11

**Authors:** Kei Kudo, Takehiro Nishimura, Ikuko Kozone, Junko Hashimoto, Noritaka Kagaya, Hikaru Suenaga, Haruo Ikeda, Kazuo Shin-ya

**Affiliations:** 1 National Institute of Advanced Industrial Science and Technology (AIST), 2-4-7 Aomi, Koto-ku, Tokyo, Japan; 2 Technology Research Association for Next Generation Natural Products Chemistry, 2-4-7 Aomi, Koto-ku, Tokyo, Japan; 3 Japan Biological Informatics Consortium (JBIC), 2-4-32 Aomi, Koto-ku, Tokyo, Japan; 4 Kitasato Institute for Life Sciences, Kitasato University, 1-15-1 Kitasato, Minami-ku, Sagamihara, Kanagawa Japan; 5 Biotechnology Research Center, The University of Tokyo, 1-1-1 Yayoi, Bunkyo-ku, Tokyo, Japan

**Keywords:** Genetic engineering, Natural products, Biosynthesis, Natural products

## Abstract

Engineering polyketide synthases is one of the most promising ways of producing a variety of polyketide derivatives. Exploring the undiscovered chemical space of this medicinally important class of middle molecular weight natural products will aid in the development of improved drugs in the future. In previous work, we established methodology designated ‘module editing’ to precisely manipulate polyketide synthase genes cloned in a bacterial artificial chromosome. Here, in the course of investigating the engineering capacity of the rapamycin PKS, novel rapamycin derivatives **1**–**4**, which lack the hemiacetal moiety, were produced through the heterologous expression of engineered variants of the rapamycin PKS. Three kinds of module deletions in the polyketide synthase RapC were designed, and the genetically engineered vectors were prepared by the in vitro module editing technique. *Streptomyces avermitilis* SUKA34 transformed with these edited PKSs produced new rapamycin derivatives. The planar structures of **1**–**4** established based on 1D and 2D NMR, ESI–TOF–MS and UV spectra revealed that **2** and **3** had skeletons well-matched to the designs, but **1** and **4** did not. The observations provide important insights into the mechanisms of the later steps of rapamycin skeletal formation as well as the ketone-forming oxygenase RapJ.

## Introduction

Engineering the biosynthetic enzymes that produce middle molecular weight (500–2000 Da) natural products, such as polyketides, is a powerful tool to generate new derivatives difficult to access through conventional synthesis^1^. Recently, we have established gene manipulating methodology for large and highly repetitive genes, especially for type I modular polyketide synthases (PKSs), in a highly precise manner^2^. In general, PKSs are comprised of multiple functional modules, and each is responsible for a C2-unit elongation of the growing polyketide chain. In a typical module, an acyltransferase (AT) domain specifically loads a malonyl- or methylmalonyl-extender unit onto an acyl carrier protein (ACP) domain. A ketosynthase (KS) domain catalyzes decarboxylative condensation between the extender unit and the growing chain, which is loaded by an ACP domain from the previous module. The nascent β-keto intermediate is optionally modified by ketoreductase (KR) domain, KR-dehydratase (DH) didomain or KR-DH-enoylreductase (ER) tridomain to give β-hydroxy, α,β-olefinic or β-methylene substructure, respectively. The domain organization of each module encoded in the biosynthetic gene(s) corresponds to the chemical structure of the secondary metabolite, which is refered to as collinearity. Thus, a genetic modification including the deletion, insertion and replacement within a module of interest alters the function of the PKS if the overall system accepts the edit^2–4^. The genetically engineered host, *Streptomyces avermitilis* SUKA, which does not produce any endogenous secondary metabolites^5,6^, can be used to readily evaluate whether genetic modifications of target PKS genes have resulted in the production of desired natural product derivatives^2^. In the course of investigating the flexibility of the rapamycin PKS, we constructed three module-deleted genes to remove the six-membered hemiacetal ring moiety of rapamycin. Rapamycin is not only clinically used as an immunosuppressant, but also known as an antitumoral and anti-aging agent, that attracts continuous interest in the development of this natural product. Studies on the crystal structure and structure–activity-relationship revealed that rapamycin binds to FK506 binding protein 12 (FKBP12) through the triketo pipecolyl substructure and recruits the mechanistic target of rapamycin (mTOR) to form a ternary complex (FKBP12-rapamycin-mTOR) to result in the inhibition of mTOR signalling pathway. To our knowledge, studies on the removal of the hemiacetal substructure of rapamycin have not been conducted. On the other hand, the smaller and structurally related analogues of rapamycin, such as nocardiopsins, show FKBP12-binding properties even though they do not possess any hemiacetal rings^7^. Together, the hemiacetal-less derivatives of rapamycin may provide important information for the structure–activity–relationships of this class of bioactive natural products. This paper describes the genetic manipulation of the rapamycin PKS genes and the fermentation, isolation, structural elucidation and biological activities of newly generated rapamycin derivatives.

## Results

### Construction of module-edited gene clusters

**Figure 1:**
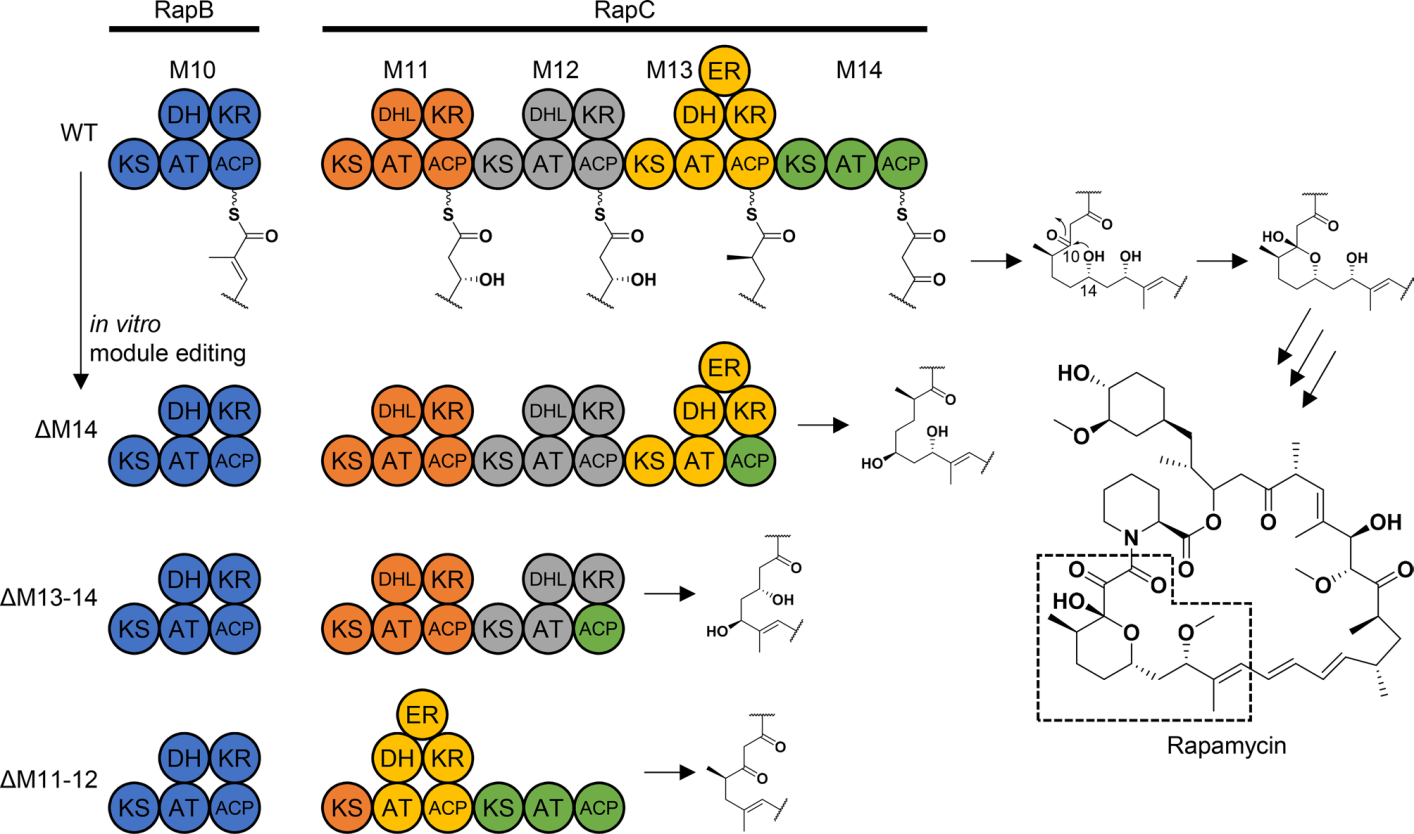
Domain organizations of PKSs edited to produce hemiacetal-less rapamycin derivatives. The circles represent each domain and are colored based on the traditional module boundary (start before KS and end after ACP). The polyketide substructure proposed to be biosynthesised by each engineered PKS is shown. For clarity, modules 5 to 9 of RapB are omitted. We achieved ΔM14 and ΔM13-14 by setting the editing point at the KR-ACP linker region, whereas the KS-AT linker region was used for ΔM11-12. Unlike the wild-type product of RapC, the designed intermediates are supposed to lack the hydroxyl or the ketone for forming the six-membered hemiacetal ring. *KS* ketosyntase, *AT* acyltransferase, *ACP* acyl carrier protein, *KR* ketoreductase, *DH* dehydratase, *DHL* dehydratase-like (inactive), *ER* enoylreductase. Dashed box corresponds to the partial structures proposed to be biosynthesized by each construct.

The experimental strategy to remove the hemiacetal substructure from rapamycin is summarized in Fig. 1. Three PKS polypeptidess, RapA, RapB and RapC, biosynthesize the polyketide backbone of rapamycin, and the non-ribosomal peptide synthetase RapP incorporates a pipecolic acid (Supplementary Fig. S1). RapP also catalyses macro-cyclization, leading to the production of pre-rapamycin (Supplementary Fig. S1). It is proposed that the hydroxyl group of C-14 attacks the C-10 ketone to form a hemiacetal ring of rapamycin (Fig. 1). Thus, we focused on the deletion of the module(s) responsible for the C-10 ketone or its δ-hydroxyl, which could yield hemiacetal-less rapamycin derivatives. Based on the functional predictions of each module in RapC, the deletion of module 14 (ΔM14), modules 13 and 14 (ΔM13-14) and modules 11 and 12 (ΔM11-12) were selected as targets. Because the linker region between a KR domain and an ACP is tolerant for deleting the modules of the rapamycin PKS^8^, we followed this strategy to construct the ΔM14 and ΔM13-14 vectors. In addition, by preserving the ACP domain and the following docking domain of module 14 protein–protein interaction between RapC and RapP are not expected to be disrupted. Based on the sequence alignment of the regions between KR and ACP domains, we set the upstream cutting site three residues after the Arg-Arg-Ala-Ala sequence conserved at the C-terminal end of the KR domains of the rapamycin PKSs (Supplementary Fig. S2). As well, the downstream cutting site was set six residues after the Gln-Arg-Tyr-Trp (the conserved LPTYxFxxxxxW motif of the post-AT linker) sequence (Supplementary Fig. S2). The junctions of the resulting module-deleted constructs are listed in Supplementary Table S1. In constructing the ΔM11-12 vector, the difficulty in genetic manipulation lies between the *rapB* and *rapC* genes because they are not consecutively located in the biosynthetic gene cluster (Supplementary Fig. S1). To avoid this, we applied another strategy that uses the linker region between a KS and an AT domain as an editing point (Supplementary Fig. S2). This strategy follows the recently suggested updated module hypothesis^9,10^, which has been shown to work well in the generation of hybrid PKSs employing the venemycin and pikromycin PKSs^4^. The protein–protein interaction between RapB and RapC, mediated by the docking domains located at the C-terminal and N-terminal ends of each protein, is preserved in this case.

In vitro module editing was carried out on pKU503rap (GenBank accession number: LC566301.1) to obtain a module-deleted construct. The original BAC was digested by Cas9 with three pairs of designed sgRNAs (Supplementary Table S2) to give linearised dsDNA. The digested BAC vector was successfully ligated to each donor DNA fragment by Gibson assembly (Supplementary Table S3), yielding pKU503rapΔM14, pKU503rapΔM13-14 and pKU503rapΔM11-12, respectively.

### Heterologous production of hemiacetal-less rapamycin derivatives

The three vectors were introduced into *S. lividans* TK24 carrying the SAP1 vector. Subsequently, the SAP1 vectors harbouring the edited gene clusters were transferred by conjugation to *S. avermitilis* SUKA34::rapH, which harbours an extra copy of the positive transcriptional regulator *rapH* in its genome to maximize production^2,11^. The transformants were cultured as described previously^2^, and we analyzed the production of rapamycin derivatives using UPLC-TOF–MS (Supplementary Figs. S3–S5). Consequently, in all three cases, peaks showing the characteristic UV absorbance at 280 nm from the triene moiety were observed. The products, **1** from ∆M14, **2** from ∆M13–14 and **3** from ∆M11–12, were isolated from the ethyl acetate extracts of the fermentation broth by the sequential chromatography on silica gel column MPLC, gel filtration and preparative reversed-phase HPLC. The ΔM11-12 construct also produced **4** as an unexpected congener, as shown below (Fig. 3). The yields of four new compounds were 0.69 ± 0.19, 3.84 ± 1.27, 3.04 ± 1.47 and 1.18 ± 0.59 mg/L, respectively, which were equivalent to 8, 45, 35 and 14% of the rapamycin yield (8.6 mg/L)^2^, respectively.

### Structural determination

**Figure 2:**
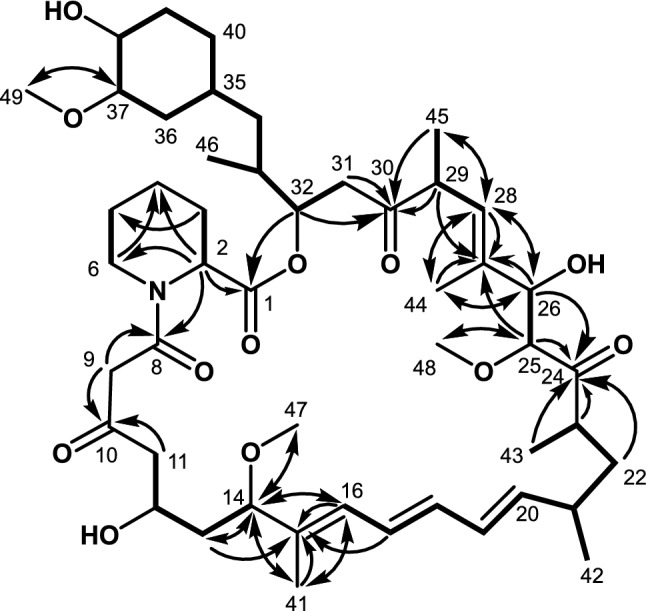
2D NMR analysis of **1**. Bold lines and arrows show DQF-COSY and HMBC correlations, respectively.

The molecular formula of **1** was established to be C_48_H_75_NO_12_ through an [M+Na]^+^ ion at *m/z* 880.5192 (calcd for C_48_H_76_NO_12_Na^+^, 880.5181) observed by high-resolution electrospray ionization mass spectrometry (HR–ESI–MS; Supplementary Fig. S3), which indicated the loss of a C3 unit from rapamycin (C_51_H_79_NO_13_). The structure **1** was established on the basis of a series of 2D NMR analyses, including DQF-COSY, HSQC and HMBC, together with the comparison of ^1^H and ^13^C chemical shift values^12^ and 2D NMR correlations with those of rapamycin. The tabulated ^1^H and ^13^C chemical shift values are shown in Table 1. Analyses of DQF-COSY revealed six ^1^H spin sequences, most of which were identical to those of rapamycin. The sequence from methylene protons 31-H_2_ (*δ*_H_ 2.92, 2.64) to methylene protons 40-H_2_ (*δ*_H_ 1.72, 0.92) through an oxymethine proton 32-H (*δ*_H_ 5.20), a methine proton 33-H (*δ*_H_ 1.97), which was, in turn ^1^H spin-coupled to methyl protons 46-H_3_ (*δ*_H_ 0.86), methylene protons 34-H_2_ (*δ*_H_ 1.12, 1.06), a methine proton 35-H (*δ*_H_ 1.42), methylene protons 36-H_2_ (*δ*_H_ 2.07, 0.65), an oxymethine proton 37-H (*δ*_H_ 2.90, *δ*_C_ 84.3), an oxymethine proton 38-H (*δ*_H_ 3.28, *δ*_C_ 73.9) and methylene protons 39-H_2_ (*δ*_H_ 1.86, 1.27) was observed. ^1^H spin couplings among an olefin proton 28-H (*δ*_H_ 5.21), a methine proton 29-H (*δ*_H_ 3.53) and methyl protons 45-H_3_ (*δ*_H_ 0.974) and between oxymethine protons 25-H (*δ*_H_ 3.96) and 26-H (*δ*_H_ 4.10) revealed a propyl and 1,2-dihydroxy ethyl moieties, respectively. A chain substructure involving a triene moiety was established by the ^1^H sequence from an olefin proton 16-H (*δ*_H_ 6.18) to methyl protons 43-H_3_ (*δ*_H_ 0.967) through olefin protons 17-H (*δ*_H_ 6.51), 18-H (*δ*_H_ 6.29), 19-H (*δ*_H_ 6.24) and 20-H (*δ*_H_ 5.50), a methine proton 21-H (*δ*_H_ 2.29), which showed a correlation with methyl protons 42-H_3_ (*δ*_H_ 1.02), methylene protons 22-H_2_ (*δ*_H_ 1.52, 1.16) and a methine proton 23-H (*δ*_H_ 2.73). A strange substructure, which does not exist in rapamycin, was established as a 1,3-dihydroxybutyl moiety by the sequence from methylene protons 11-H_2_ (*δ*_H_ 2.67) to an oxymethine proton 14-H (*δ*_H_ 3.82) through an oxymethine proton 12-H (*δ*_H_ 4.04) and methylene protons 13-H_2_ (*δ*_H_ 1.70, 1.65). Finally, the remaining spin coupling systems between an α-methine proton 2-H (*δ*_H_ 5.19, *δ*_C_ 51.7) and methylene protons 3-H_2_ (*δ*_H_ 1.72, 0.92) and between methylene protons 5-H_2_ (*δ*_H_ 1.59, 1.41) and nitrogen-bonded methylene protons 6-H_2_ (*δ*_H_ 3.59, 2.97, *δ*_C_ 43.9) established two ethyl moieties, which are members of a pipecolic acid substructure, as shown below (Fig. 2).

**Table 1:** ^13^C and ^1^H NMR spectroscopic data for **1** in acetone-*d*_6_ (150/600 MHz, respectively).

No	*δ*_C_	*δ*_H_ (multiplicity, *J* in Hz)
1	177.2	
2	51.7	5.19 (m)
3	26.0	2.23 (d, 13.7), 1.53 (m)
4	20.5	1.67 (m), 1.37 (m)
5	25.1	1.59 (m), 1.41 (m)
6	43.9	3.59 (d, 12.7), 2.97 (dd, 3.0, 13.0)
8	166.6	
9	50.5	3.86 (d, 16.3), 3.52 (d, 16.3)
10	203.6	
11	50.1	2.67 (m)
12	65.4	4.04 (m)
13	41.0	1.70 (m), 1.65 (m)
14	85.0	3.82 (q, 7.4)
15	136.7	
16	128.9	6.18 (d, 11)
17	127.2	6.51 (dd, 11.2, 13.9)
18	132.7	6.29 (dd, 10.7, 14.0)
19	131.1	6.24 (dd, 10.2, 14.3)
20	139.5	5.50 (dd, 9.4, 14.1)
21	36.0	2.29 (m)
22	39.5	1.52 (m), 1.16 (m)
23	40.9	2.73 (m)
24	211.3	
25	85.2	3.96 (d, 6.4)
26	77.4	4.10 (d, 5.6)
27	137.6	
28	126.7	5.21 (d, 4.4)
29	46.0	3.53 (m)
30	207.7	
31	40.4	2.92 (m), 2.64 (m)
32	74.0	5.20 (m)
33	32.9	1.97 (m)
34	39.5	1.12 (m), 1.06 (m)
35	33.1	1.42 (m)
36	35.5	2.07 (m), ovl; 0.65 (q, 11.6)
37	84.3	2.90 (m), ovl
38	73.9	3.28 (m)
39	32.3	1.86 (m), ovl; 1.27 (m)
40	31.0	1.72 (m), 0.92 (m)
41	9.7	1.68 (s)
42	21.3	1.02 (d, 6.5)
43	13.4	0.967 (d, 6.5)
44	12.2	1.85 (s)
45	14.8	0.974 (d, 6.5)
46	15.3	0.86 (d, 6.8)
47	55.0	3.12 (s)
48	57.1	3.23 (s)
49	56.2	3.35 (s)

The connectivity among these substructures was established by ^1^H–^13^C long-range couplings in the HMBC spectrum. According to the gene modification of the rapamycin biosynthetic gene cluster, **1** could have pipecolic acid moiety, but its six-membered ring structure could not be confirmed by DQF-COSY due to signal overlaps. ^1^H–^13^C long-range couplings from the α-methine proton 2-H and the methylene protons 6-H_2_ to a methylene carbon C-3 (*δ*_C_ 32.3) and from the α-methine proton 2-H to the nitrogen-bonded methylene carbon C-6 and an ester carbonyl carbon C-1 (*δ*_C_ 177.2) confirmed the existence of a pipecolic acid moiety. HMBC correlations from the methylene protons 31-H_2_ and the methyl proton 45-H_3_ to a carbonyl carbon C-30 (*δ*_C_ 207.7), from the singlet methyl protons 44-H_3_ (*δ*_H_ 1.85) to olefinic carbons C-27 (*δ*_C_ 137.6), C-28 (*δ*_C_ 126.7) and an oxymethine carbon C-26 (*δ*_C_ 77.4), from the oxymethine proton 25-H and the methyl protons 43-H_3_ to a carbonyl carbon C-24 (*δ*_C_ 211.3) and from the singlet methyl protons 41-H_3_ (*δ*_H_ 1.68) to an oxymethine carbon C-14 (*δ*_C_ 85.0), olefinic carbons C-15 (*δ*_C_ 136.7) and C-16 (*δ*_C_ 128.9) elucidated the ^13^C sequence from C-11 to C-40. ^1^H–^13^C long-range couplings from the methylene protons 11-H_2_ and the methylene protons 9-H_2_ (*δ*_H_ 3.59, 2.67) to a carbonyl carbon C-10 (*δ*_C_ 203.6), from the methylene protons 9-H_2_ and the α-methine proton 2-H to an amide carbonyl carbon C-8 (*δ*_C_ 166.6) and from the oxymethine proton 32-H to a carbonyl carbon C-1 established the main skeletal ring structure of **1**. Three methoxy protons, 47-H (*δ*_H_ 3.12), 48-H (*δ*_H_ 3.23) and 49-H (*δ*_H_ 3.32), are long-range coupled to C-14, C-25 (*δ*_C_ 85.2) and C-37 (*δ*_C_ 84.3), which proved the substitution carbons of these methoxy groups. Overall, the structure including stereochemistry deduced from the interpretation of the biosynthetic gene cluster of **1** was determined, as shown in Fig. 2. The structure was not expected to be produced from the modified biosynthetic gene cluster (∆M14) of rapamycin (see Fig. 1). In particular, according to the sequence of the AT domain of module 13, **1** should possess a methyl group at the C-9 position.

**Figure 3:**
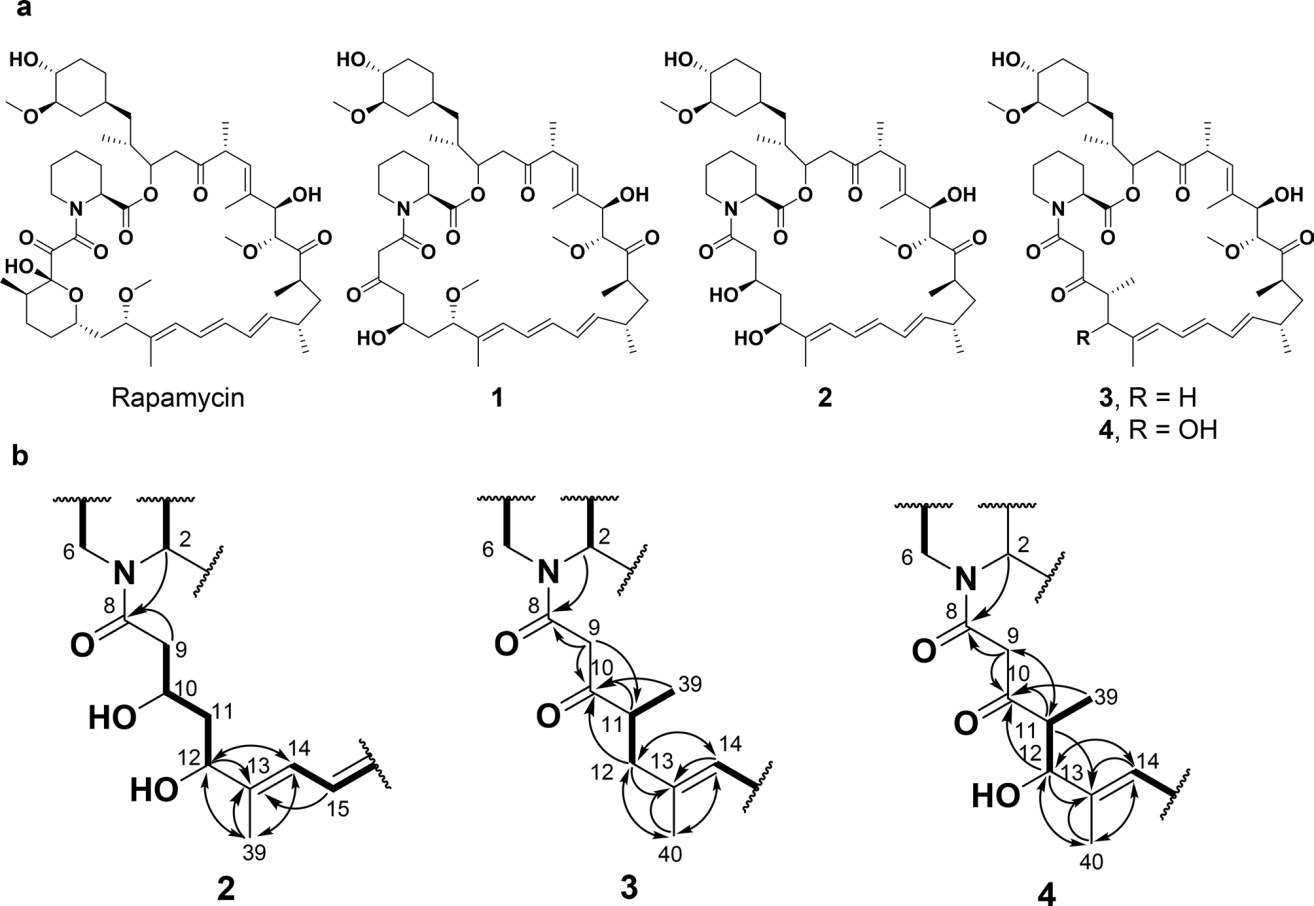
Structural determination of newly isolated hemiacetal-less rapamycin derivatives. (**a**) Structures of rapamycin and compounds **2**–**4**. The stereochemistry of the derivatives is deduced from that of rapamycin as well as the stereoselectivities of corresponding tailoring domains. (**b**) Selected ^1^H–^1^H DQF-COSY and HMBC correlations in **2**–**4**. Bold lines and arrows show DQF-COSY and HMBC correlations, respectively.

Compound **2**, which was obtained from SUKA34::rapH/pKU503rapΔM13-14, has a molecular formula of C_45_H_71_NO_11_ determined by an [M+Na]^+^ ion at *m/z* 824.4913 (calcd for C_45_H_71_NO_11_Na^+^, 824.4919) in an HR–ESI–MS (Supplementary Fig. S4). Analyses of the 2D NMR spectra of **2** revealed that the skeletal structure from a triene moiety to a cyclohexyl moiety together with a pipecolic acid moiety in **2** is the same as that of rapamycin, as shown in Supplementary Fig. S6. The changed structure was established as follows: the sequence from methylene protons 9-H_2_ (*δ*_H_ 2.51, 2.46) to an oxymethine proton 12-H (*δ*_H_ 4.37) through an oxymethine proton 10-H (*δ*_H_ 4.01) and methylene protons 11-H_2_ (*δ*_H_ 1.70, 1.64) was observed in the DQF-COSY spectra. ^1^H–^13^C long-range couplings from an α-methine proton 2-H (*δ*_H_ 5.13, *δ*_C_ 52.5) and the methylene protons 9-H_2_ to an amide carbonyl carbon C-8 (*δ*_C_ 173.1) revealed the connectivity between the pipecolic acid and a 1,3-dihydroxybutyl moiety, as shown in Fig. 3. In addition, HMBC correlations from singlet methyl protons 39-H_3_ (*δ*_H_ 1.75) to an oxymethine carbon C-12 (*δ*_C_ 76.9), olefinic carbons C-13 (*δ*_C_ 141.2) and C-14 (*δ*_C_ 126.5) established the linkage between C-12 and C-14. Thus, the 27-membered structure of **2** was elucidated as shown in Fig. 3.

The module-edited construct pKU503rap∆M11-12 produced two compounds, **3** and **4**. The molecular formula of **3** was established as C_46_H_71_NO_10_ from an ion at *m*/*z* 820.4980 [M+Na]^+^ (calcd for C_46_H_71_NO_10_Na^+^, 820.4970) by positive ion HR–ESI–MS data (Supplementary Fig. S5). As in the case of compounds **1** and **2**, the partial structures of **3** from a triene moiety to a cyclohexyl moiety and a pipecolic acid moiety are equivalent to that of rapamycin, as shown in Supplementary Fig. S6. The sequence from methyl protons 39-H_3_ (*δ*_H_ 1.14) to methylene protons 12-H_2_ (*δ*_H_ 2.39, 2.21) through a methine proton 11-H (*δ*_H_ 3.06) indicated the presence of a propyl moiety. In the HMBC spectrum of **3**, the ^1^H–^13^C long-range couplings from an α-methine proton (*δ*_H_ 5.24) in the pipecolic acid and methylene protons 9-H_2_ (*δ*_H_ 3.72, 3.63) to an amide carbonyl carbon C-8 (*δ*_C_ 166.6), from the methylene protons 9-H_2_ and the methyl protons 39-H_3_ to another carbonyl carbon C-10 (*δ*_C_ 207.4) and from methyl protons 40-H_3_ (*δ*_H_ 1.81) to methylene carbon C-12 (*δ*_C_ 42.4), a quaternary olefin carbon C-13 (*δ*_C_ 136.5) and olefinic methine carbon C-14 (*δ*_C_ 126.2) determined the structure of **3**, as shown in Fig. 3.

The molecular formula of **4** was determined by HR–ESI–MS to be C_46_H_71_NO_11_ (found: 836.4904 [M+Na]^+^, calculated for C_46_H_71_NO_11_Na^+^, 836.4919), which indicated that **4** is an oxidized compound of **3** (Supplementary Fig. S5). Analyses of a variety of NMR spectra revealed that the basic skeletal structure is almost identical to that of **3** (Supplementary Fig. S6). The structural difference between **3** and **4** is recognised at the position of C-12 (in **3**, *δ*_H_ 2.39, 2.21, *δ*_C_ 42.4). The methylene C-12 was replaced by an oxymethine (*δ*_H_ 4.24, *δ*_C_ 79.2) in **4**.

### Biological activity

**Figure 4:**
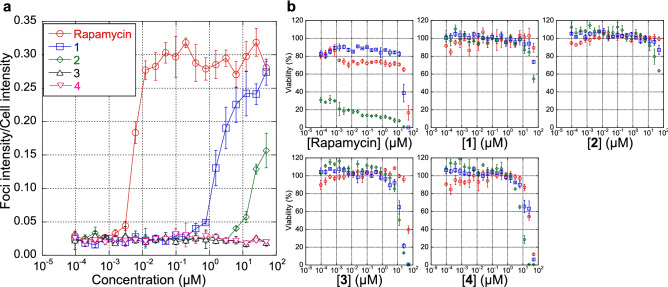
Biological activities. (**a**) Binding activities of rapamycin and its derivatives to FKBP and mTOR. (**b**) cytostatic activities of rapamycin and its derivatives to SKOV-3 (red), MESO-1 (blue) and Jurkat (green) cells. Each plot with error bars indicates average ± s.d. calculated from three independent biological replicates.

To evaluate the importance of the hemiacetal moiety of rapamycin on its biological activity, we examined the activities of **1**–**4** to stabilize the binding between FKBP12 and mTOR by employing the Fluoppi (Fluorescent based protein–protein interactions) system^13^. As a result, **1** showed ~ 1000 times weaker activity (EC_50_ = 1.88 µM) than that of rapamycin (5.6 nM) (Fig. 4a). Furthermore, **2** showed much less activity, and **3** and **4** showed no detectable activity (Fig. 4a). In comparison with the other 29-membered derivatives with a hemiacetal ring that did not reduce the activity so dramatically (~ 10% reduction versus rapamycin)^2^, these results indicate that changes in the hemiacetal and also in the diketo-moiety prevent efficient formation of the ternary complex with FKBP12 and mTOR.

Rapamycin shows cytostatic effects against human ovarian adenocarcinoma SKOV-3 cells, malignant pleural mesothelioma MESO-1 cells and T lymphoma Jurkat cells (Fig. 4b). In contrast, **3** and **4** did not show cytostatic effects but rather cytotoxic activities at relatively high concentrations, while both cytostatic and cytotoxic activities were abolished in **1** and **2**. Cytotoxicities at a high concentration were also observed in the case of rapamycin.

## Discussion

Hemiacetal-less rapamycin derivatives were successfully produced through one- or two-module deletions within RapC. As with previous reports^2,8^, the module deletion strategy performed well in the rapamycin PKS system. The skeletons of **2** and **3** were matched the designs well, while **1** and **4** did not.

A possible interpretation of the production of the demethylated- and oxo-derivative **1** can be considered from three aspects: the substrate specificity of RapP, the extender unit specificity of the last AT domain and the functionality of the reductive loop. First, RapP may gatekeep against the α-methyl intermediate (Supplementary Fig. S7), while β-hydroxyl or γ-methyl groups are allowed (cf. **2**–**4**). This hypothesis also suggests that the formation of a hemiacetal ring occurs after RapP-catalyzed macrocyclisation. Second, if the AT domain of module 13 (AT13) is promiscuous in selecting an extender unit, it can cause malonyl-CoA incorporation into the growing polyketide chain. The relaxed specificity of AT domains has also been observed in an engineered erythromycin PKS^14^ and in AT8 of the spinosyn PKS^15^. Alternatively, if ACP14 cannot interact with AT13, AT12 may load malonyl-CoA onto ACP14 to yield **1**. The production of **2** by the ΔM13-14 construct demonstrates the sufficient interaction between ACP14 and AT12. There is also a possibility that the native malonyl-CoA:ACP transacylase from primary metabolism overrides AT13 as observed for an AT6-inactivated erythromycin PKS^16^. Third, the interaction between ACP14 and KR13 could be insufficient to process the β-oxo-group. Dysfunctionality of KR domains in engineered contexts has been reported in three-module systems^17,18^. Concerning substrate specificity, we can also predict that RapP prefers the β-oxo-intermediate to the β-methylene pattern. A relatively low yield of **1** (8% that of rapamycin) compared to **2** (45%) and **3** + **4** (49% combined) may reflect the lower efficiency of these shunt processes.

The hydroxyl group at C-12 in **4** could be explained by the impaired function of the DH domain or the promiscuity of a P450 enzyme, such as RapJ, which catalyzes the successive oxidation at C-9 of rapamycin to form the diketo-moiety^19^. Interestingly, all four hemiacetal-less derivatives obtained in this study lack the C-9 keto group even though a rapamycin derivative formed through the deletion of five modules and possessing a strange ester bond is reported to have the C-9 keto group^8^. Although further enzymological evidence is required, this study provides important insights to understand the catalytic capability of RapJ.

The diminished biological activities of hemiacetal-less rapamycin derivatives were almost as expected. The crystal structures revealed that the hemiacetal moiety interacts with the hydrophobic cavity of FKBP^20,21^ and the triketo pipecolyl moiety was shown to be essential for binding to FKBP^22^. However, the fact that **1** retained the very weak ability to form the FKBP-**1**-mTOR ternary complex indicates that even the hemiacetal- and diketo-less macrocyclic skeleton could promote or inhibit some protein–protein interactions.

## Methods

### Bacterial strains and culture conditions

Bacterial strains and culture conditions reported in our previous work were used^2^. The growth of *Escherichia coli* strains DH5α, NEB10β (New England Biolabs Japan Inc., Tokyo, Japan) and GM2929 *hsdS*::Tn*10*^5^ was achieved using LB containing 10 g of tryptone, 5 g of yeast extract and 5 g of NaCl in 1 L of deionized water adjusted to pH 7.5, and the solid medium (LA) was prepared by adding 15 g of agar to 1 L of LB medium. In the production of rapamycin derivatives, *Streptomyces avermitilis* SUKA carrying the biosynthetic gene cluster for each derivative was inoculated into a 50-mL test tube with 15 mL of GSY medium containing 5 g of glucose, 15 g of soya flour and 5 g of yeast extract per liter of deionized water, and the cells were cultured with reciprocal shaking at 320 rpm at 27 °C for 2 days. A 375 μL aliquot of the vegetative culture was used to inoculate a 125-mL flask containing 15 mL of production medium containing 40 g of β-cyclodextrin, 20 g of Pharmamedia, 5 g of glycerol, 21.2 g of MES, 5 mg of ZnSO_4_·7H_2_O, 5 mg of CuSO_4_·5H_2_O and 5 mg of MnCl_2_·4H_2_O per liter of deionized water adjusted to pH 6.0. The fermentation culture was shaken at 24 °C for 5 days at 180 rpm. Large-scale preparations of the products were carried out in a baffled 500-mL flask containing 100 mL of the production medium.

### Introduction of edited BACs into *S. avermitilis* SUKA

The procedure follows that of our previous work^2^. All clones were introduced into *S. lividans* TK24 carrying the SAP1 vector (containing the synthetic sequence of *attB*_*ϕK38-1*_, *attB*_*R4*_, *attB*_*ϕBT1*_, *attB*_*ϕC31*_ and *attB*_*TG1*_ of *S. avermitilis* MA-4680). All clones were integrated into the SAP1 vector because the bacteriophage attachment sites (*attB*) were located in the SAP1 vector but not in the chromosome. The transfer of the SAP1 vector containing the edited gene cluster for rapamycin was conducted as described^23^. The objective transconjugants were confirmed by the antibiotic-resistance phenotype and the size of the linear plasmid by contour-clamped homogeneous electric field (CHEF)^24^ electrophoresis using SAP1::pKU503rap as the control linear plasmid.

### In vitro module editing of the rap gene cluster on BAC

The experimental procedure followed a previously described method^2^. The sequences of oligonucleotides and donor DNA fragments are listed in Supplementary Tables S2 and S3, respectively. pKU503rap (GenBank accession number: LC566301.1) was digested with Cas9 and a pair of sgRNA in vitro, and the resulting linear dsDNA and corresponding donor DNA fragment were joined by Gibson assembly. Transformants were screened by PCR using corresponding primers (Supplementary Table S2).

### UPLC/UV/MS analysis of the metabolites from the transformant

A 750 μL aliquot of the culture was extracted with 750 μL of *n*-butanol (*n*-BuOH), and the organic layer was subjected to the UPLC/UV/MS analysis. The analytical procedure follows our previous work^2^. Analytical UPLC and HR–ESI–MS (positive mode) were performed using a Waters ACQUITY UPLC System (Waters, Taunton, MA) in conjunction with a BEH ODS column (2.1 i.d. × 100 mm, Waters), a Waters ACQUITY UPLC photodiode array eλ detector (Waters) and a XevoG2 ToF system (Waters). Mobile phase A was water + 0.1% formic acid, and mobile phase B was acetonitrile + 0.1% formic acid. The elution program was 5–100% B over 5 min and 100% B for 1 min at a flow rate of 0.8 mL min^−1^. Data acquisitions and analyses were performed using MassLynX V4.1 software (Waters). The yields were calculated from the peak area of UV absorption chromatogram at 280 nm. A standard curve was prepared using rapamycin.

### Isolation of compound 1

The procedure follows our previous work^2^. Compound **1** was isolated from SUKA34::rapH/pKU503rapΔM14. Six liters of fermentation broth of SUKA34::rapH/pKU503rapΔM14 were centrifuged to obtain a mycelial cake, which was extracted twice with 750 mL of acetone. The acetone was removed *in vacuo*, and the residual aqueous layer was extracted three times with ethyl acetate (EtOAc). The resultant EtOAc layer was concentrated *in vacuo* to afford 3.91 g of crude extract. The crude extract was subjected to medium-pressure liquid chromatography (MPLC) on silica gel (SNAP Ultra 25 g, Biotage, Uppsala, Sweden) eluted with a gradient system of *n*-hexane–EtOAc (0–25% EtOAc) followed by a stepwise solvent system of chloroform (CHCl_3_)–methanol (MeOH) (0, 1, 3, 5, 10, 50 and 90% MeOH). The 3% and 5% MeOH fractions (875 mg) were combined with the EtOAc extract of the broth centrifugal supernatant (2.05 g) and subjected to silica gel MPLC (SNAP Ultra 25 g) with isocratic elution with 4% MeOH in CHCl_3_. The fractions were monitored by TLC and UPLC analyses, and fractions containing **1** (145 mg) were collected and further purified by Sephadex LH-20 column chromatography (1:1 CHCl_3_/MeOH) to obtain a fraction containing **1** (29.4 mg). Finally, the sample containing **1** was purified by preparative reversed-phase HPLC using a CAPCELL PAK MG-II C18 column (5.0 μm, 20 i.d. × 150 mm; Shiseido, Tokyo, Japan), and the elution with 50% aqueous acetonitrile supplemented with 0.1% formic acid yielded 2.5 mg of **1** as a colourless oil and as a 4:1 mixture of rotamers. The assignments of ^1^H and ^13^C NMR signals are noted in Table 1.

### Isolation of compound 2

The procedure follows our previous work^2^. Compound **2** was isolated from SUKA34::rapH/pKU503rapΔM13-14. Two liters of fermentation broth of SUKA34::rapH/pKU503rapΔM13-14 were centrifuged to obtain a mycelial cake, which was extracted twice with 250 mL of acetone. The acetone was removed *in vacuo*, and the residual aqueous layer was extracted three times with ethyl acetate (EtOAc). The resultant EtOAc layer was concentrated *in vacuo* to afford 1.23 g of crude extract. The crude extract was subjected to medium-pressure liquid chromatography (MPLC) on silica gel (SNAP Ultra 25 g, Biotage, Uppsala, Sweden) eluted with a gradient system of *n*-hexane–EtOAc (0–25% EtOAc) followed by a stepwise solvent system of chloroform (CHCl_3_)–methanol (MeOH) (0, 1, 3, 5, 10, 50 and 90% MeOH). The 3% and 5% MeOH fractions (173 mg) were combined with the EtOAc extract of the broth centrifugal supernatant (286 mg) and subjected to silica gel MPLC (SNAP Ultra 25 g) with isocratic elution with 4% MeOH in CHCl_3_. The fractions were monitored by TLC and UPLC analyses, and fractions containing **2** (23.7 mg) were collected and further purified by Sephadex LH-20 column chromatography (1:1 CHCl_3_/MeOH) to obtain a fraction containing **2** (23.7 mg). Finally, the sample containing **2** was purified by preparative reversed-phase HPLC using a CAPCELL PAK MG-II C18 column (5.0 μm, 20 i.d. × 150 mm; Shiseido, Tokyo, Japan), and the elution with 50% aqueous acetonitrile supplemented with 0.1% formic acid yielded 1.3 mg of **2** as a colorless, amorphous solid and as a 4:1 mixture of rotamers. The assignments of ^1^H and ^13^C NMR signals are noted in Supplementary Table S4.

### Isolation of compounds 3 and 4

The procedure follows our previous work^2^. Compounds **3** and **4** were isolated from SUKA34::rapH/pKU503rapΔM11-12. Six liters of fermentation broth of SUKA34::rapH/pKU503rapΔM11-12 were centrifuged to obtain a mycelial cake, which was extracted twice with 750 mL of acetone. The acetone was removed *in vacuo*, and the residual aqueous layer was extracted twice with ethyl acetate (EtOAc). The resultant EtOAc layer was concentrated *in vacuo* to afford 2.67 g of crude extract. The crude extract was subjected to medium-pressure liquid chromatography (MPLC) on silica gel (SNAP Ultra 25 g, Biotage, Uppsala, Sweden) eluted with a gradient system of *n*-hexane–EtOAc (0–25% EtOAc) followed by a stepwise solvent system of chloroform (CHCl_3_)–methanol (MeOH) (0, 1, 3, 5, 10, 50 and 90% MeOH). The 3% MeOH fraction (405 mg) was combined with the EtOAc extract of the broth centrifugal supernatant (757 mg) and subjected to silica gel MPLC (SNAP Ultra 25 g) with isocratic elution with 4% MeOH in CHCl_3_. The fractions were monitored by TLC and UPLC analyses, and fractions containing **3** (137 mg) and **4** (85.9 mg) were collected, respectively. The sample containing **3** was purified by Sephadex LH-20 column chromatography (1:1 CHCl_3_/MeOH) to obtain a fraction containing **3** (34.1 mg). Finally, purification by preparative reversed-phase HPLC using a CAPCELL PAK MG-II C18 column (5.0 μm, 20 i.d. × 150 mm; Shiseido, Tokyo, Japan) and the elution with 60% aqueous acetonitrile supplemented with 0.1% formic acid yielded 9.8 mg of **3** as a white, amorphous solid as a 4:1 mixture of rotamers. Similarly, the sample containing **4** was purified by Sephadex LH-20 column chromatography (1:1 CHCl_3_/MeOH) to obtain a fraction containing **4** (13.0 mg). Finally, purification by preparative reversed-phase HPLC using a CAPCELL PAK MG-II C18 column (5.0 μm, 20 i.d. × 150 mm; Shiseido, Tokyo, Japan) was performed, and the elution with 40% aqueous acetonitrile supplemented with 0.1% formic acid yielded 1.9 mg of **4** as a white amorphous solid as a 4:1 mixture of rotamers. The assignments of ^1^H and ^13^C NMR signals are noted in Supplementary Table S5.

### Binding activities of rapamycin derivatives to FKBP and mTOR

The procedure is same as our previous work^2^. HeLa cells were maintained in DMEM (Fujifilm Wako, Osaka, Japan) supplemented with 10% FBS (Thermo Fisher Scientific, MA, USA) and 1% of Penicillin–Streptomycin (Thermo Fisher Scientific) in a humidified incubator with 5% CO_2_ at 37 °C. Harvested cells were plated at 1 × 10^6^ cells/well in a 6-well plate and incubated for 6 h. Each 1.25 µg of phAG-mTOR and pAsh-FKBP12 (Medical & Biological Laboratories Co., Ltd., Aichi, Japan) was co-transfected with Lipofectamine 2000 reagent (Thermo Fisher Scientific) overnight. After overnight incubation, transfected cells were harvested and seeded in a 384-well optical-bottom microplate (PerkinElmer, MA, USA) at a density of 3000 cells/well. After the 24-h incubation at 37 °C, cells were treated with different concentrations of compounds for 1 h. Cells were then fixed with 4% paraformaldehyde and stained with 1 µg/ml of Hoechst33342 (Thermo Fisher Scientific). Cell images of 9 visual fields per well were acquired with a × 20 objective lens using an Opera Phenix High-Content Screening System (PerkinElmer) and were analysed using Harmony4.9 software (PerkinElmer). Because protein–protein interactions are quantified by measuring the aggregation of fluorescent elements in foci, the fluorescent intensity in foci was normalized by the total fluorescence intensity in each cell. EC_50_ values were calculated using TIBCO Spotfire 10.3 software (PerkinElmer).

### Cytotoxicity assay

The procedure follows our previous work^2^. The cytotoxic activities of isolated rapamycin derivatives against T lymphoma Jurkat cells, human ovarian adenocarcinoma SKOV-3 cells and malignant pleural mesothelioma MESO-1 cells were examined. Jurkat cells were cultured in RPMI1640 medium supplemented with 10% foetal bovine serum, penicillin (50 U/mL), streptomycin (50 μg/mL) and Glutamax. SKOV-3 cells were cultured in DMEM medium supplemented with 10% fetal bovine serum, penicillin (50 U/mL) and streptomycin (50 μg/mL). MESO-1 cells were cultured in RPMI1640 medium supplemented with 10% fetal bovine serum, penicillin (50 U/mL) and streptomycin (50 μg/mL). All cell lines were seeded in a 384-well plate at a density of 1000 cells/well in 20 μL of media and were incubated at 37 °C in a humidified incubator with 5% CO_2_. After 4 h of incubation, samples diluted twofold in DMSO were added to the cell culture at a concentration of 0.5% (0.1 μL) and were incubated for 72 h. Cell viabilities were measured using a CellTiter-Glo luminescent cell viability assay and an EnVision multilabel plate reader.

## Supplementary Information

Supplementary Information 1.

Supplementary Information 2.

## Data Availability

All data generated or analyzed during this study are included in this published article and its Supplementary Information file.
