# Supplementary material for: Hemiacetal-less rapamycin derivatives designed and produced by genetic engineering of a type I polyketide synthase
Source: Sci Rep. 2021 May 11;11:9944. doi: 10.1038/s41598-021-88583-z (PMC8113240; doi:10.1038/s41598-021-88583-z)
Supplement: Supplementary file 1 — Supplementary Information 1. [file 41598_2021_88583_MOESM1_ESM.docx]

Hemiacetal-less rapamycin derivatives designed and produced by genetic engineering of a type I polyketide synthase

Supplementary information

Kudo et al.

Figures S1-S7

Tables S1-S3

Supplementary Data (NMR data) 1-20


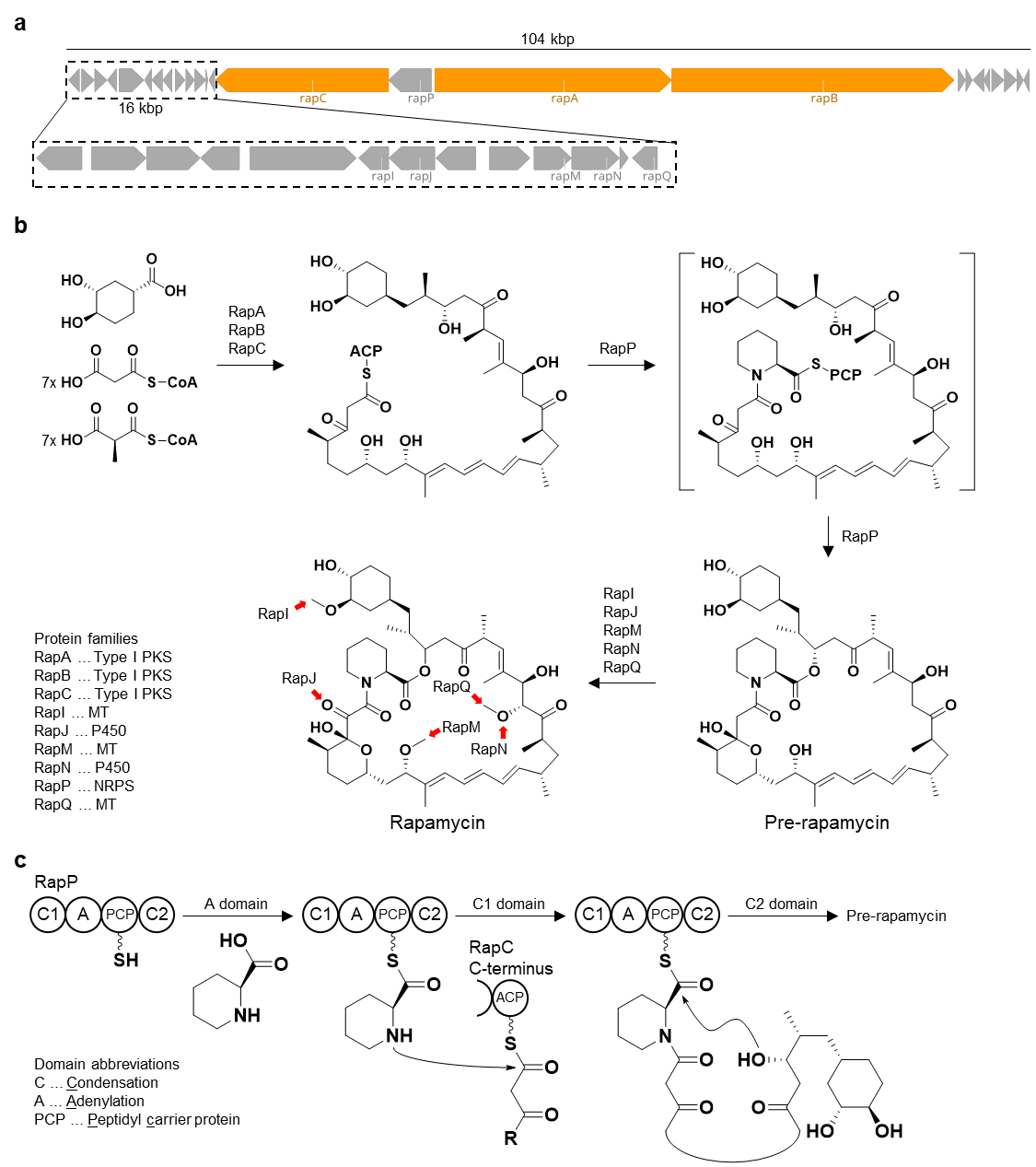


**Figure S1**. Rapamycin biosynthesis. **a**, Schematic view of the biosynthetic gene cluster. Arrows colored orange are the genes encoding type I PKSs, and those colored grey are the other biosynthetic genes. **b**, Summary of the biosynthetic pathway to rapamycin. Three PKSs and one non-ribosomal peptide synthetase (NRPS) assemble the macrocyclic intermediate, pre-rapamycin. The tailoring enzymes of which red arrows indicate the corresponding modifications sequentially process pre-rapamycin to give rapamycin. **c**, The predicted function of RapP. The first condensation (C) domain (C1) catalyzes the incorporation of a pipecolic acid unit into the growing polyketide chain passed from the C-terminus ACP domain of RapC. The second C domain (C2) catalyzes intramolecular macrocyclization to release pre-rapamycin.


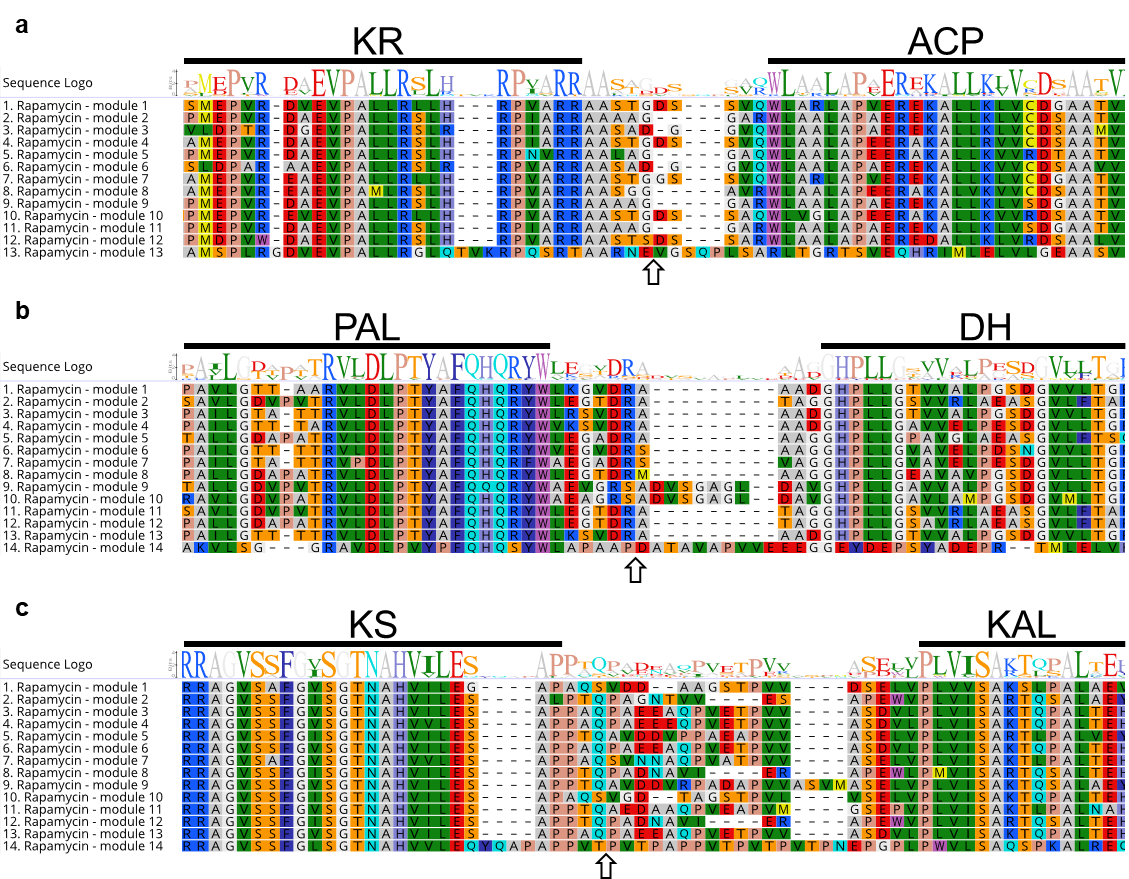


**Figure S2**. Alignment analyses of rapamycin PKS modules. Arrows indicate the editing points used in this study. To delete module 14 or modules 13-14, the upstream editing point was set at the flexible region between KR and ACP (panel **a**), while the downstream one was set between PAL (post-AT linker) and DH (panel **b**). Note that the aligned sequence of module 14 in panel b is the region between AT and ACP. To delete modules 11-12, the flexible region between KS and KAL (KS-AT linker) was selected (panel **c**). The resulting chimera sequences are listed in Supplementary Table S1.


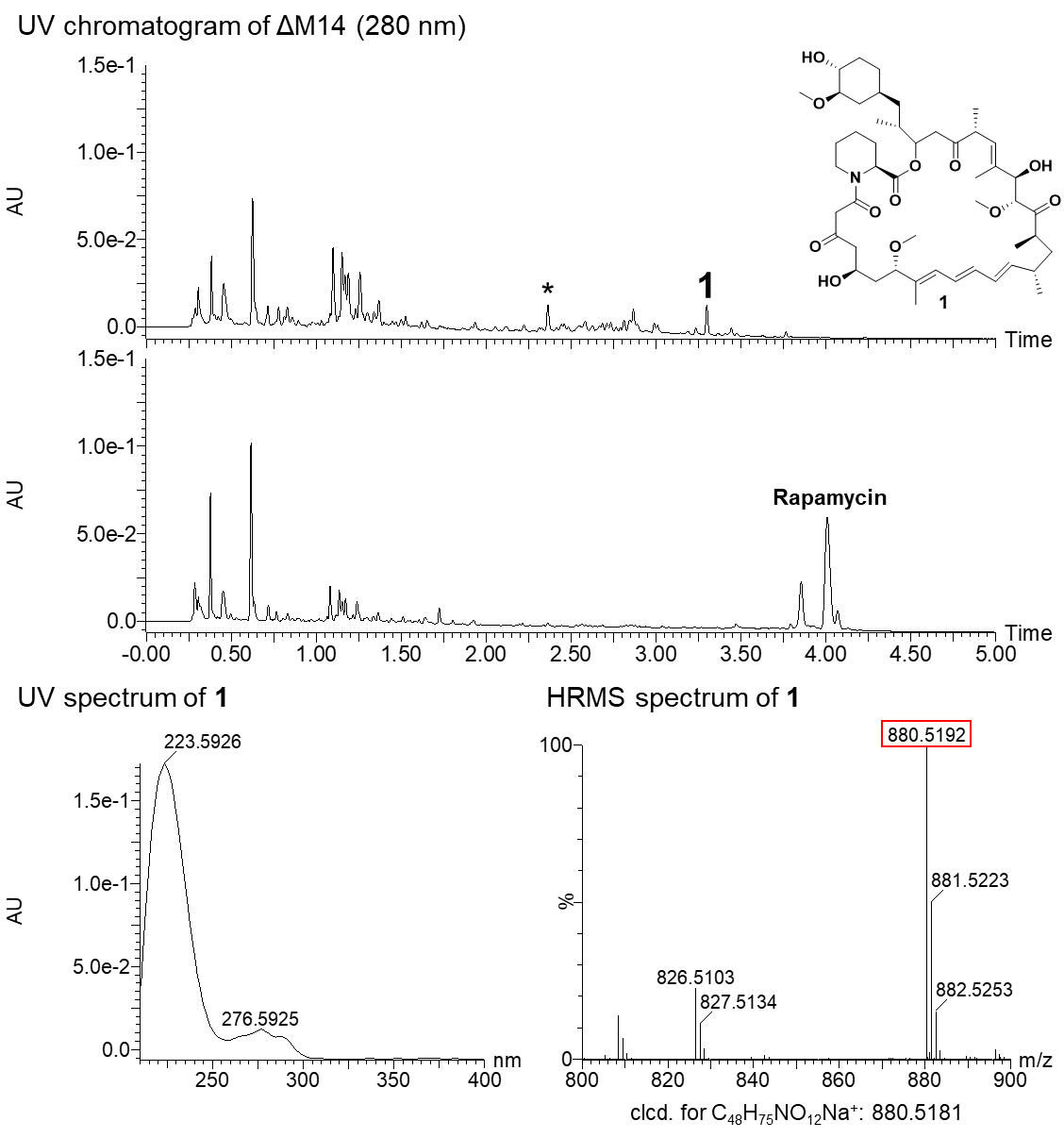


**Figure S3**. UPLC-TOF-MS analysis of *S. avermitilis* SUKA34::rapH/pKU503rapΔM14. The UV absorption chromatogram was aligned with that of *S. avermitilis* SUKA34::rapH/pKU503rap (a transformant carrying unmodified BAC clone). The asterisk indicates a putative shunt product which can be observed in other transformants carrying different constructs. Data is the representative of five biological replicates.


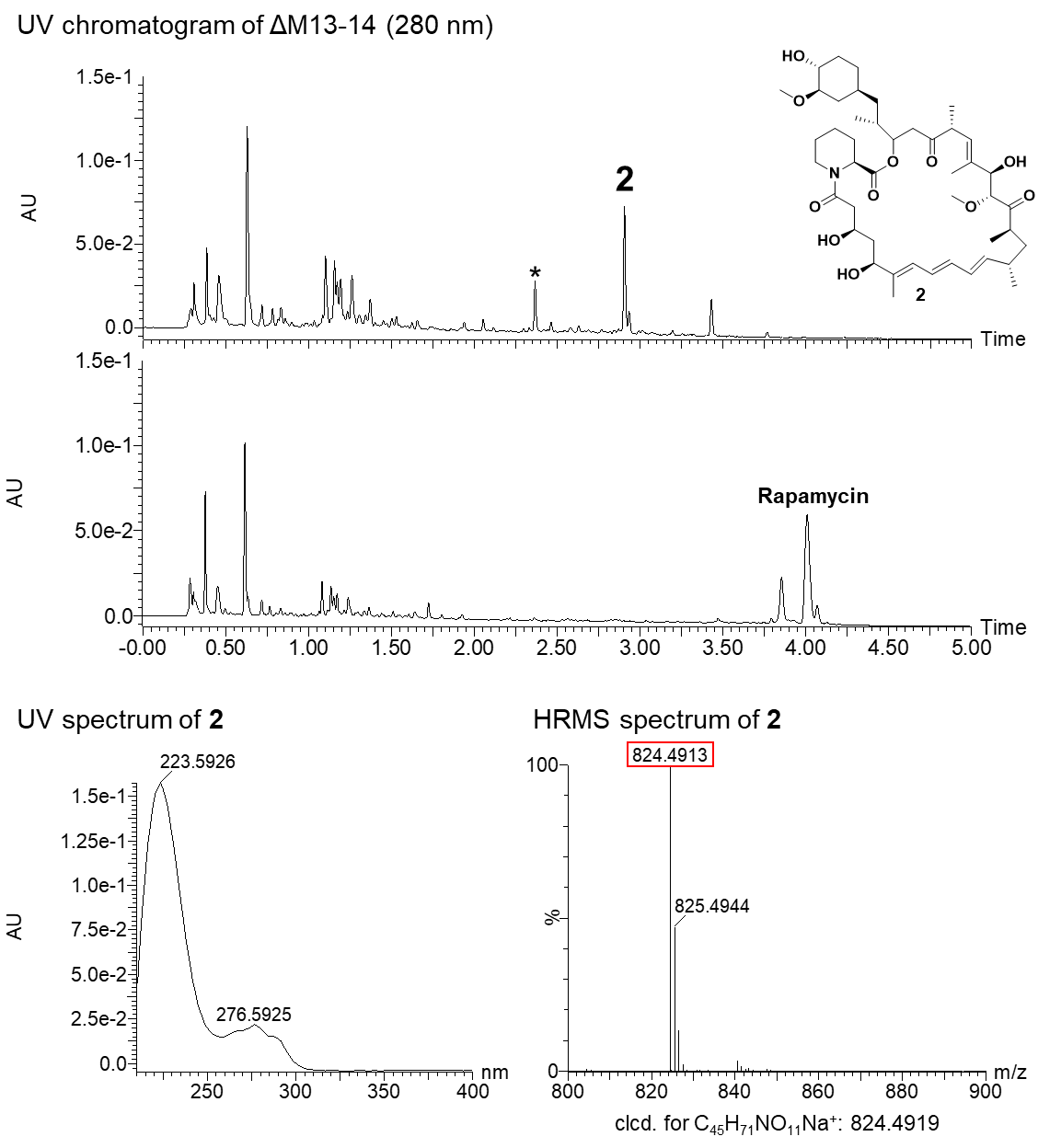


**Figure S4**. UPLC-TOF-MS analysis of *S. avermitilis* SUKA34::rapH/pKU503rapΔM13-14. The UV absorption chromatogram was aligned with that of *S. avermitilis* SUKA34::rapH/pKU503rap (a transformant carrying unmodified BAC clone). The asterisk indicates a putative shunt product which can be observed in other transformants carrying different constructs. Data is the representative of five biological replicates.


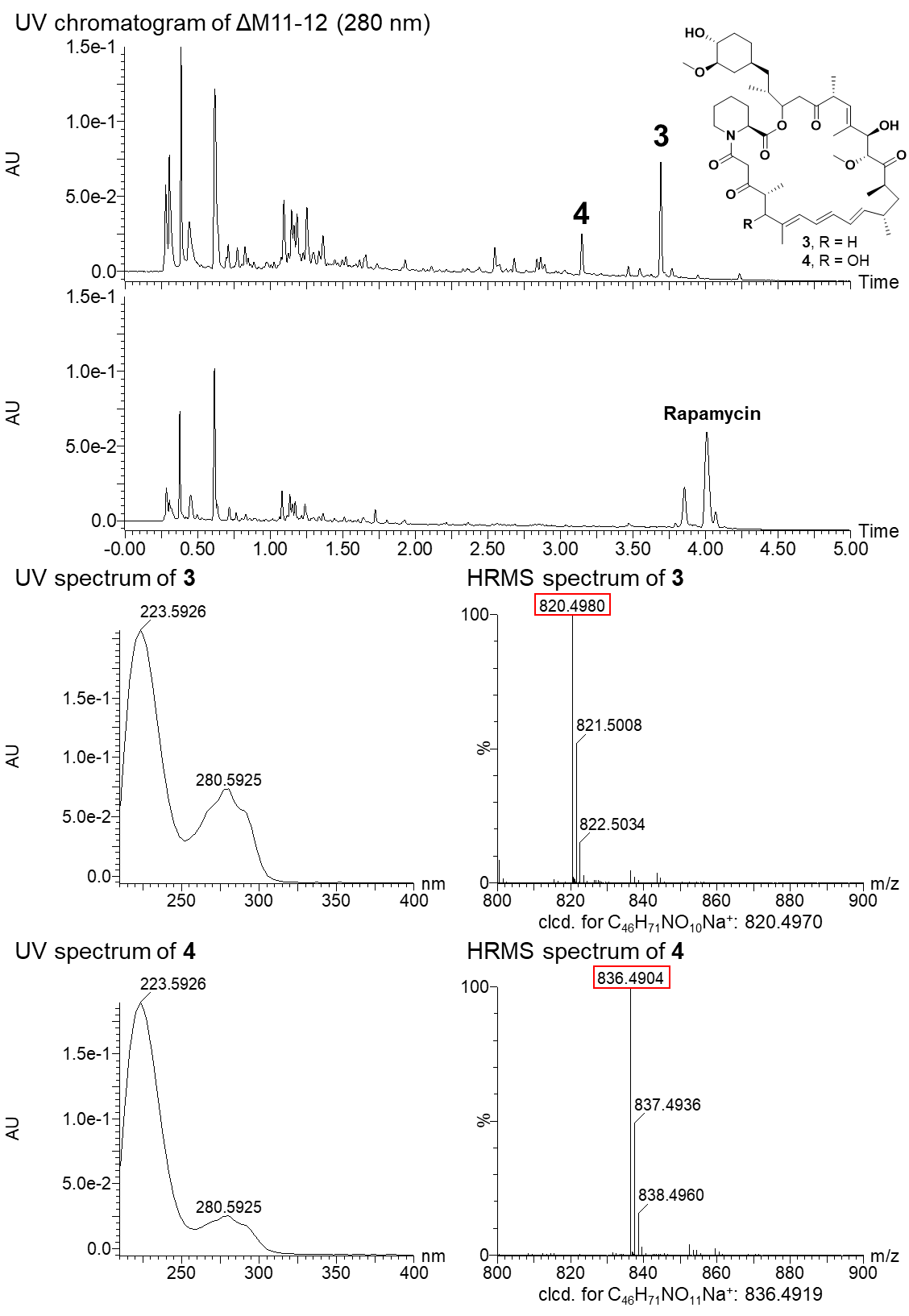


**Figure S5**. UPLC-TOF-MS analysis of *S. avermitilis* SUKA34::rapH/pKU503rapΔM11-12. The UV absorption chromatogram was aligned with that of *S. avermitilis* SUKA34::rapH/pKU503rap (a transformant carrying unmodified BAC clone). Data is the representative of five biological replicates.


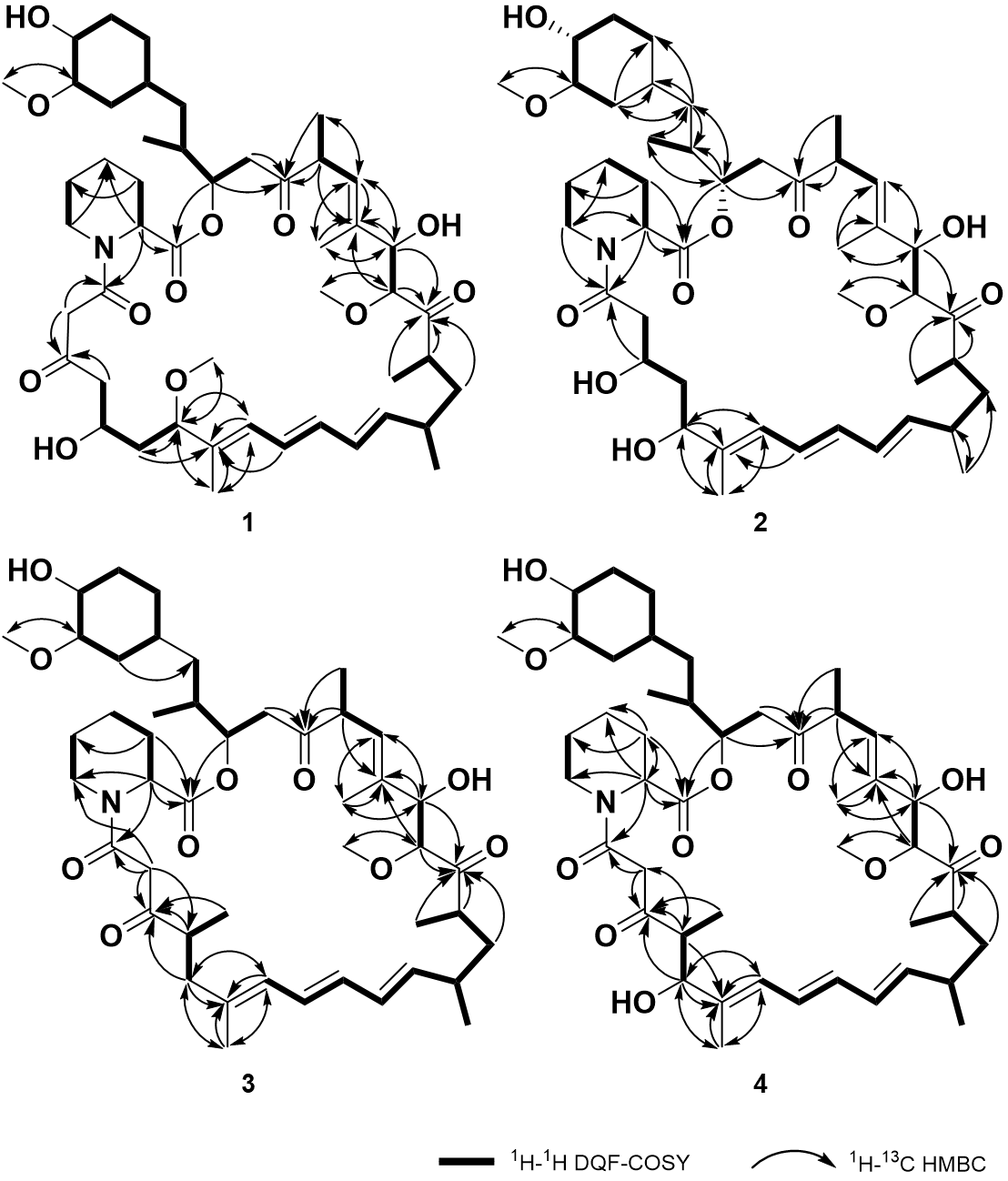


**Figure S6**. Selected ^1^H–^1^H DQF-COSY and HMBC correlations in **1**–**4**.


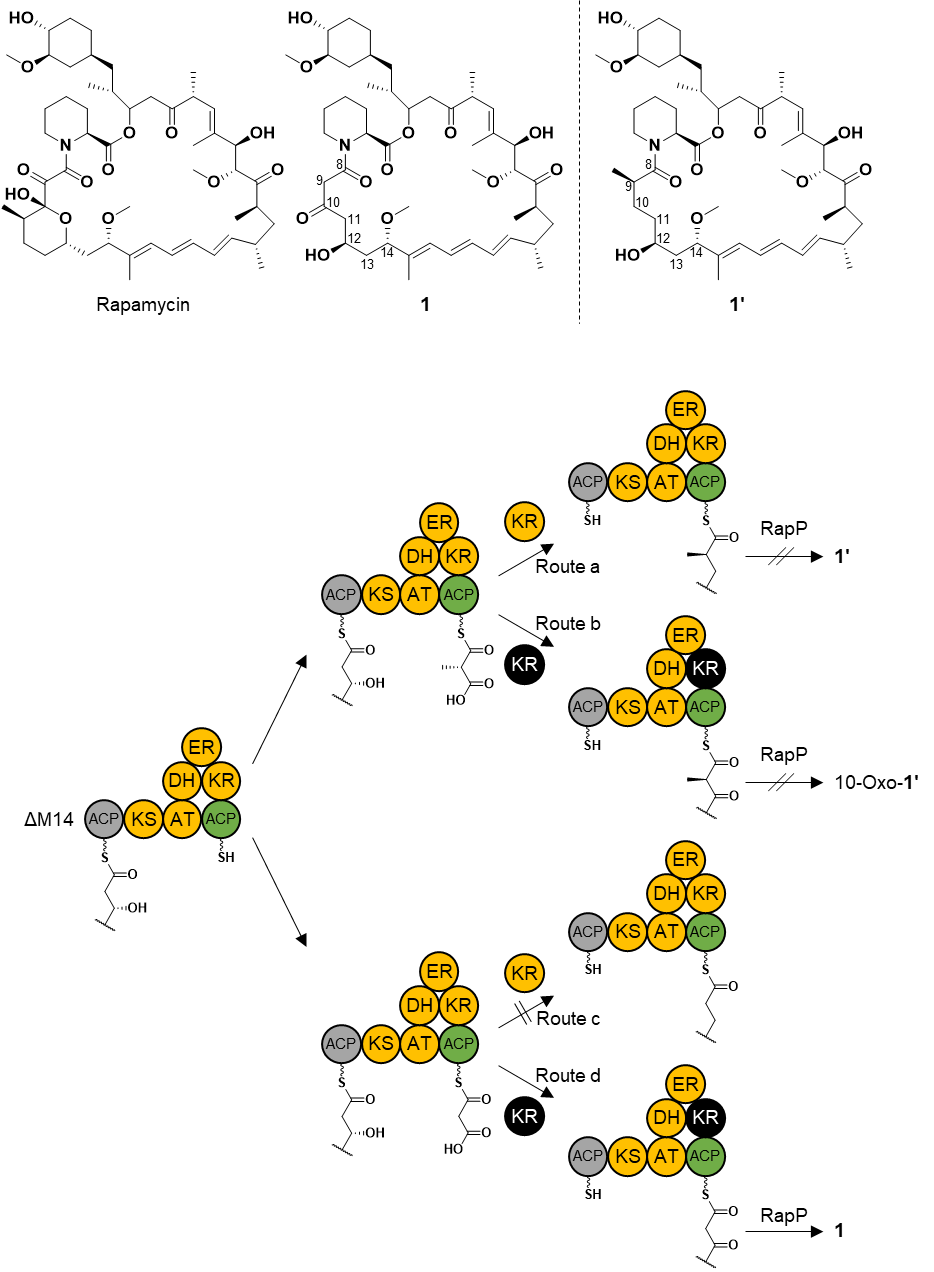


**Figure S7**. The structural comparison of **1** and anticipated compound **1’**. Route a, the anticipated route at first, does not proceed due to the possible gatekeeping function of RapP. Route b as well as Route d illustrates the case that KR13 does not act (black circles), but 10-oxo-**1**’ was not detected. Route c was a possible route if KR13 functioned on a malonyl-CoA incorporated intermediate. Route d is the proposed biosynthetic route to **1**.

**Table S1.** The amino acid sequences of edited modules used in this study.

| Construct | Sequence (N-term -> C-term) | Location of the editing point | Produced compound(s) |
| --- | --- | --- | --- |
| ΔM14 | EQRRAEGLPA LALAWGLWDD TSGLTSGLTD TDHDRIRRSG  MRTITAEHGM RLFDGASRHG EPVLFAAAMS PLRGDVEVPA  LLRGLQTVKR PQSRTAARNE/DATAVAPVVE EEGGEYDEPS  YADEPRTMLE LVHMEVASLL GMADPGVILD DSSFLELGFD  SLSAVRLRNR LSKATGLDLP STLLFEHPTS AELAAHLDAL | between the KR of module 13 and the ACP of module 14 | **1** |
| ΔM13-14 | DALAEERRAE GLPALAVAWG LWEDASGLTT QLTDTDRDRI  RRGGLRTITA EYGMRLFDTA SRHGNPILVA APMDPVWDAE  VPALLRSLHR PVARRAASTS/DATAVAPVVE EEGGEYDEPS  YADEPRTMLE LVHMEVASLL GMADPGVILD DSSFLELGFD  SLSAVRLRNR LSKATGLDLP STLLFEHPTS AELAAHLDAL | between the KR of module 12 and the ACP of module 14 | **2** |
| ΔM11-12 | DREQPLLLGS LKSNIGHTQA AAGVSGVIKM VMALRHALVP  RTLHVDEPSR HVDWTAGAVE LVTENQPWPE IGRPRRAGVS  SFGVSGTNAH VILESAPPTQ/PAEEAQPVET PVVASDVLPL  VISAKTQPAL TEHEDRLRAY LAASPGADIR AVASTLAVTR  SVFEHRAVLL GDDTVTGTAV TDPRIVFVFP GQGWQWLGMG | between the KS of module 11 and the AT of module 13 | **3** and **4** |

Slashes (“/”) indicate the editing point of each construct.

One hundred residues upstream and downstream of the editing point are shown, respectively.

**Table S2.** Oligonucleotides used in this study.

| Name | Sequence (5’ -> 3’) | Description |
| --- | --- | --- |
| ΔM14_5’_sgRNA | TTCTAATACGACTCACTATAGACCTCGACGTCCCCCCGTAGGTTTTAGAGCTAGA | template for sgRNA synthesis  (ΔM14) |
| ΔM14_3’_sgRNA | TTCTAATACGACTCACTATAGCGGGGCTCATCCGCATAGGAGTTTTAGAGCTAGA | template for sgRNA synthesis  (ΔM14) |
| ΔM13-14_5’_sgRNA | TTCTAATACGACTCACTATAGCGCACCGATGGACCCGGTTTGTTTTAGAGCTAGA | template for sgRNA synthesis  (ΔM13-14) |
| ΔM13-14_3’_sgRNA | TTCTAATACGACTCACTATAGCGGGGCTCATCCGCATAGGAGTTTTAGAGCTAGA | template for sgRNA synthesis  (ΔM13-14) |
| ΔM11-12_5’_sgRNA | TTCTAATACGACTCACTATAGAAGTGCTTCCAGTTTGGCGCGTTTTAGAGCTAGA | template for sgRNA synthesis  (ΔM11-12) |
| ΔM11-12_3’_sgRNA | TTCTAATACGACTCACTATAGCGCTGCCGGAGTCTGACACCGTTTTAGAGCTAGA | template for sgRNA synthesis  (ΔM11-12) |
| rap_M13KR_3'_Fw | GACACCAGCGGCCTCACCTCCGGG | primer for PCR screening  (ΔM14, ΔM13-14) |
| rap_M13ACP_3'_Rv | AGATGCGCGGCCAGAGCGTTCGCC | primer for PCR screening  (ΔM14, ΔM13-14) |
| rap_M12DH_3'_Fw | GAAACCCCCGCCGCGTGGCAGGCGC | primer for PCR screening  (ΔM11-12) |
| rap_M12DH-KR_Rv | ACCGCTTCAGGCTCAGACCCACTG | primer for PCR screening  (ΔM11-12) |

**Table S3.** Donor DNAs used in this study.

| Name | Sequence (5’ -> 3’) | Description |
| --- | --- | --- |
| ΔM14_donor | CGGTGAGCCGGTCCTGTTCGCCGCGGCGATGTCTCCGCTACGGGGGGACGTCGAGGTCCCCGCGCTGCTGCGCGGACTTCAAACGGTGAAGCGTCCGCAGTCCAGGACCGCGGCACGCAACGAGGATGCCACAGCTGTGGCGCCCGTGGTCGAGGAGGAGGGCGGCGAGTATGACGAGCCCTCCTATGCGGATGAGCCCCGCACCATGCTGGAACTCGTTCACA | donor DNA for Gibson assembly  (ΔM14) |
| ΔM13-14_donor | CATGGCAACCCGATTCTGGTCGCCGCACCGATGGACCCGGTTTGGGACGCGGAAGTCCCCGCGCTCCTCCGCTCGTTGCATCGTCCCGTCGCCCGGCGGGCCGCCTCTACCAGCGATGCCACAGCTGTGGCGCCCGTGGTCGAGGAGGAGGGCGGCGAGTATGACGAGCCCTCCTATGCGGATGAGCCCCGCACCATGCTGGAACTCGTTCACA | donor DNA for Gibson assembly  (ΔM13-14) |
| ΔM11-12_donor | TATCTTCGCTGGGCGACTGCGGAGTTGCACACCACCCGCGCCAAACTGGAAGCACTTGCCGCCGCCAACACCGAGCCGTTGGCGATTGTGGGTATGGCGTGCCGTCTGCCGGGTGGGGTGTCGTCGCCGGAGGATCTGTGGCGCTTGGTCGAGTCGGGTACGGACGCGATTTCCGACTTCCCCGCCGACCGTGGCTGGGACGTCGAGAACCTGTACGACCCTGATCCGGACGCGTCCGGGAAGTCGTACTGCGTGCAGGGTGGCTTCCTGGACTCCGCAGGCGGTTTCGACGCCTCGTTCTTCGGGATCAGCCCGCGTGAGGCGTTGGCGATGGATCCACAGCAGCGACTGGTCCTGGAGGTGTCCTGGGAGGCGTTCGAACGGGCCGGGATCGAGCCCGGTTCCCTTCGCGGCAGCGACACCGGCGTCTTCATAGGCGCTTACCCAGGCGGCTACGGCGCCGGTGCCGGTGCTGACCTTGAGGGTTATGGCACCACATCCGGTCCCAGCGTGCTCTCCGGCCGGGTGTCGTACTTCTTCGGCCTCGAAGGCCCCGCCATCACGGTGGACACCGCATGTTCTTCGTCGCTGGTGGCGTTGCATCAGGCGGGGTACGCCCTGCGGCAGGGGGAATGTTCCCTGGCCCTGGTCGGCGGTGTCACCGTGATGGCCACGCCGGACGTCTTCACCGAATTCGCCCGGCAGCGTGGCCTGGCCACCGACGGCCGCAGCAAAGCCTTCGCGGACAGTGCGGACGGTGCCGGATTCTCCGAGGGCATCGGTGTGCTGCTGGTGGAGCGTTTGTCGGACGCTGAGGCCAAGGGTCACCAGGTGCTGGCGGTGGTCCGCAGTTCGGCGGTCAACCAGGACGGCGCGTCCAACGGCCTGACCGCGCCGAACGGCCCCTCCCAGCAGCGAGTGATCCAGACCGCGCTCAGCAACGCCGGTTTGACCACAGCCGAGGTGGACGTGGTCGAGGGCCACGGCACGGGTACGACGCTGGGCGACCCGATCGAAGCCCAAGCCGTGATCGCCACCTACGGCCAGGACCGTGAACAGCCCCTGCTGCTCGGATCGCTGAAATCAAACATCGGTCATACCCAGGCCGCCGCTGGCGTATCGGGTGTCATCAAGATGGTGATGGCCCTGCGGCATGCTTTGGTGCCGCGTACGTTGCATGTGGATGAGCCGTCGCGGCATGTGGACTGGACGGCGGGTGCGGTTGAGCTGGTGACGGAAAACCAGCCGTGGCCGGAGATTGGCCGGCCGCGCCGGGCAGGCGTGTCCTCCTTTGGAGTCAGTGGCACTAATGCCCACGTCATCCTGGAGAGCGCACCCCCCACGCAGCCCGCGGAGGAGGCGCAGCCTGTTGAGACGCCGGTGGTGGCCTCGGATGTGCTGCCGCTGGTGATATCGGCCAAGACCCAGCCCGCCCTGACCGAACACGAAGACCGGCTGCGCGCCTACCTGGCGGCGTCGCCCGGGGCGGATATACGGGCTGTGGCATCGACGCTGGCGGTGACACGGTCGGTGTTCGAGCACCGCGCCGTACTCCTTGGAGATGACACCGTCACCGGCACCGCGGTGACCGACCCCAGGATCGTGTTTGTCTTTCCCGGGCAGGGGTGGCAGTGGCTGGGGATGGGCAGTGCACTGCGCGATTCGTCGGTGGTGTTCGCCGAGCGGATGGCCGAGTGTGCGGCGGCGTTGCGCGAGTTCGTGGACTGGGATCTGTTCACGGTTCTGGATGATCCGGCGGTGGTGGACCGGGTTGATGTGGTCCAGCCCGCTTCCTGGGCGATGATGGTTTCCCTGGCCGCGGTGTGGCAGGCGGCCGGTGTGCGGCCGGATGCGGTGATCGGCCATTCGCAGGGTGAGATCGCCGCAGCTTGTGTGGCGGGTGCGGTGTCACTACGCGATGCCGCCCGGATCGTGACCTTGCGCAGCCAGGCGATCGCCCGGGGCCTGGCGGGCCGGGGCGCGATGGCATCCGTCGCCCTGCCCGCGCAGGATGTCGAGCTGGTCGACGGGGCCTGGATCGCCGCCCACAACGGGCCCGCCTCCACCGTGATCGCGGGCACCCCGGAAGCGGTCGACCATGTCCTCACCGCTCATGAGGCACAAGGGGTGCGGGTGCGGCGGATCACCGTCGACTATGCCTCGCACACCCCGCACGTCGAGCTGATCCGCGACGAACTACTCGACATCACTAGCGACAGCAGCTCGCAGACCCCGCTCGTGCCGTGGCTGTCGACCGTGGACGGCACCTGGGTCGACAGCCCGCTGGACGGGGAGTACTGGTACCGGAACCTGCGTGAACCGGTCGGTTTCCACCCCGCCGTCAGCCAGTTGCAGGCCCAGGGCGACACCGTGTTCGTCGAGGTCAGCGCCAGCCCGGTGTTGTTGCAGGCGATGGACGACGATGTCGTCACGGTTGCCACGCTGCGTCGTGACGACGGCGACGCCACCCGGATGCTCACCGCCCTGGCACAGGCCTATGTCCACGGCGTCACCGTCGACTGGCCCGCCATCCTCGGCACCACCACAACCCGGGTACTGGACCTTCCGACCTACGCCTTCCAACACCAGCGGTACTGGCTCAAGAGCGTGGACCGGGCGGCTGCCGACGGTCATCCACTGCTGGGCACCGTAGTGGCACTGCCCGGCTCCGACGGTGTGGTGCTCACCGGGCGGGTGTCGCTGGCCACCCATACATGGCTGGCCGATCACGCGGTCCGGGGCAGTGTCCTGCTCCCCGGGACCGCATTTGTGGAACTGGTCGTCCGCGCCGCCGACGAGGTCGAGTGCGACGTCGTTGACGAGTTGGTGATCGAAACCCCGCTCCTGCTGCCGCAGACCGGAGGCGTCCAACTGTCCGTGTCCGTCGCCGAGGCCGACGAGTCCGGACACCGCACGGTGACGGTCTTCTCCCAGGCGGACAACACGGACGCATGGATCCGGCACGTTTCCGCCACTATCAGCACCTCTGACACACCCCTCTCGCTGCCGGAGTCTGACACCTGGCCGCCAGCCCAGGCCCAGCCGATGAACGTGGCCG | donor DNA for Gibson assembly  (ΔM11-12) |

All donor DNAs were prepared as a synthetic DNA purchased from GENEWIZ Japan Corp. (Saitama, Japan)

**Table S4.**  ^13^C and ^1^H NMR spectroscopic data for **2** in acetone-d_6_ (150/600 MHz, respectively)

| No. | *δ*_C_ | *δ*_H_ (multiplicity, *J* in Hz) |
| --- | --- | --- |
| 1 | 171.5 |  |
| 2 | 52.5 | 5.13 (3.8) |
| 3 | 27.0 | 2.16 (d, 16.5), 1.65 (m) |
| 4 | 21.2 | 1.69 (m), 1.35 (m) |
| 5 | 25.7 | 1.70 (m), 1.35 (m) |
| 6 | 43.7 | 3.78 (d, 13.2), 3.16 (dd, 12.7, 2.9) |
| 8 | 173.1 |  |
| 9 | 41.3 | 2.51 (dd, 16.1, 5.52), 2.46 (dd, 16.7, 7.49) |
| 10 | 67.8 | 4.01 (m) |
| 11 | 42.6 | 1.70 (m), 1.64 (m) |
| 12 | 76.9 | 4.37 (d, 6.6) |
| 13 | 141.2 |  |
| 14 | 126.5 | 6.06 (d, 11.3) |
| 15 | 128.4 | 6.44 (dd, 13.6, 11.2) |
| 16 | 131.8 | 6.23 (m) |
| 17 | 133.1 | 6.24 (m) |
| 18 | 139.8 | 5.47 (dd, 9.5, 13.9) |
| 19 | 36.4 | 2.29 (m) |
| 20 | 40.6 | 1.50 (m), 1.18 (m) |
| 21 | 41.5 | 2.70 (m) |
| 22 | 212.0 |  |
| 23 | 86.6 | 3.91 (d, 6.2) |
| 24 | 77.8 | 4.12 (d, 6.1) |
| 25 | 138.0 |  |
| 26 | 126.9 | 5.35 (d, 8.7) |
| 27 | 46.8 | 3.36 (q, 7.3) |
| 28 | 208.2 |  |
| 29 | 42.0 | 2.63 (m) |
| 30 | 75.3 | 5.15 (dd, 4.54, 8.6) |
| 31 | 34.0 | 1.89 (m) |
| 32 | 39.7 | 1.23 (m), 1.10 (m) |
| 33 | 34.0 | 1.44 (m) |
| 34 | 35.9 | 2.09 (m), 0.65 (q, 12.0) |
| 35 | 85.4 | 2.90, ovl |
| 36 | 74.7 | 3.28 (ddd, 11.2, 8.6, 4.7) |
| 37 | 33.2 | 1.88 (m), 1.28 (dt, 11.7, 3.2) |
| 38 | 32.4 | 1.67 (m), 0.97 (m) |
| 39 | 11.2 | 1.75 (s) |
| 40 | 22.4 | 1.01 (d,3.6) |
| 41 | 14.5 | 0.99 (d, 7.1) |
| 42 | 13.4 | 1.75 (s) |
| 43 | 16.2 | 1.02 (d, 4.0) |
| 44 | 16.0 | 0.96(d, 6.5) |
| 45 | 58.0 | 3.21 (s) |
| 46 | 57.2 | 3.34 (s) |

ovl., overlapping to the water peak.

**Table S5.** ^13^C and ^1^H NMR spectroscopic data for **3** and **4** in acetone-d_6_ (125/500 MHz, respectively)

| **3** | | | **4** | | |
| --- | --- | --- | --- | --- | --- |
| No. | *δ*_C_ | *δ*_H_ (multiplicity, *J* in Hz) | No. | *δ*_C_ | *δ*_H_ (multiplicity, *J* in Hz) |
| 1 | 170.2 |  | 1 | 171.1 |  |
| 2 | 51.7 | 5.24 (d, 5.2) | 2 | 52.2 | 5.21 (m) |
| 3 | 26.1 | 2.23 (m) | 3 | 26.5 | 2.23 (d, 14.6), 1.66 (m) |
| 4 | 20.4 | 1.69 (m) | 4 | 20.7 | 1.73 (m), 1.43 (m) |
| 5 | 25.0 | 1.60 (m) | 5 | 23.4 | 1.73 (m), 1.53 (m) |
| 6 | 44.0 | 3.53 (d, 14.9), 2.98 (dd, 12.7, 13.7) | 6 | 44.6 | 3.86 (d, 15.2), 3.26 (m) |
| 8 | 166.6 |  | 8 | 167.5 |  |
| 9 | 48.4 | 3.72 (d, 16.2), 3.63 (d, 16.2) | 9 | 50.1 | 3.87 (d, 15.1), 3.45 (d, 15.1) |
| 10 | 207.4 |  | 10 | 206.7 |  |
| 11 | 44.8 | 3.06 (q, 6.9) | 11 | 50.1 | 2.94 (m) |
| 12 | 42.4 | 2.39 (dd, 8.8, 14.6), 2.21 (dd, 3.8, 13.7) | 12 | 79.2 | 4.24 (d, 7.95) |
| 13 | 136.5 |  | 13 | 139.0 |  |
| 14 | 126.2 | 6.00 (d, 11.2) | 14 | 126.4 | 6.11 (d, 10.96) |
| 15 | 127.7 | 6.47 (dd, 11.2, 14.6) | 15 | 127.6 | 6.48 (dd, 11.3, 14.3) |
| 16 | 131.3 | 6.23 (m) | 16 | 133.1 | 6.29 (dd, 10.6, 14.2) |
| 17 | 130.7 | 6.24 (m) | 17 | 130.9 | 6.24 (dd, 10.6, 14.6) |
| 18 | 138.2 | 5.61 (dd, 8.9, 14.0) | 18 | 139.3 | 5.61 (dd, 9.0, 14.4) |
| 19 | 34.8 | 2.35 (m) | 19 | 35.2 | 2.37 (m) |
| 20 | 39.5 | 1.63 (m), 1.18 (m) | 20 | 40.0 | 1.60 (d, 15.0), 1.16 (m) |
| 21 | 41.2 | 2.68 (m) | 21 | 41.4 | 2.65 (m) |
| 22 | 210.7 |  | 22 | 210.8 |  |
| 23 | 85.3 | 4.09 (d, 5.0) | 23 | 85.7 | 4.05 (d, 4.7) |
| 24 | 76.8 | 4.21 (m) | 24 | 77.2 | 4.18 (d, 4.6) |
| 25 | 137.0 |  | 25 | 137.3 |  |
| 26 | 125.5 | 5.39 (d, 10.0) | 26 | 125.8 | 5.36 (d, 10.1) |
| 27 | 46.0 | 3.49 (m) | 27 | 46.1 | 3.48 (dq, 2.3 , 7.1) |
| 28 | 207.7 |  | 28 | 207.5 |  |
| 29 | 40.5 | 2.93 (m), 2.70 (dd, 4.1, 17.3) | 29 | 41.2 | 2.76 (dd, 5.1, 7.1) |
| 30 | 74.2 | 5.22 (m) | 30 | 74.6 | 5.19 (m) |
| 31 | 32.8 | 1.96 (m) | 31 | 33.5 | 1.94 (m) |
| 32 | 39.0 | 1.25 (m), 1.11 (m) | 32 | 39.1 | 1.24 (m), 1.11 (m) |
| 33 | 33.2 | 1.45 (m) | 33 | 33.6 | 1.45 (m) |
| 34 | 35.2 | 2.10 (m), 0.70 (q, 12.0) | 34 | 35.4 | 2.11 (m), 0.71 (q, 12.2) |
| 35 | 84.5 | 2.91 (m) | 35 | 84.9 | 2.92 (m) |
| 36 | 73.8 | 3.31 (m) | 36 | 74.2 | 3.32 (m) |
| 37 | 32.3 | 1.89 (m), 1.30 (d, 11.6) | 37 | 32.7 | 1.91 (m), 1.31 (d, 11.6) |
| 38 | 31.3 | 1.73 (m), 0.99 (m) | 38 | 31.8 | 1.78 (m), 1.00 (m) |
| 39 | 16.5 | 1.14 (d, 6.7) | 39 | 14.3 | 1.05 (d, 7.0) |
| 40 | 17.6 | 1.81 (s) | 40 | 12.7 | 1.82 (s) |
| 41 | 21.5 | 1.03 (d, 6.5) | 41 | 21.5 | 1.07 (d, 6.5) |
| 42 | 13.2 | 0.95 (d, 6.6) | 42 | 13.6 | 0.90 (d, 6.6) |
| 43 | 13.1 | 1.89 (s) | 43 | 13.5 | 1.88 (s) |
| 44 | 15.4 | 1.03 (d, 6.5) | 44 | 15.9 | 1.08 (d, 6.6) |
| 45 | 14.8 | 0.87 (d, 6.7) | 45 | 15.4 | 0.98 (d, 7.0) |
| 46 | 57.1 | 3.28 (s) | 46 | 57.3 | 3.24 (s) |
| 47 | 56.2 | 3.37 (s) | 47 | 56.6 | 3.33 (s) |

**NMR data**

**Supplementary Data 1.** ^1^H NMR spectrum (Acetone-*d_6_*, 600 MHz) of compound **1**.

**
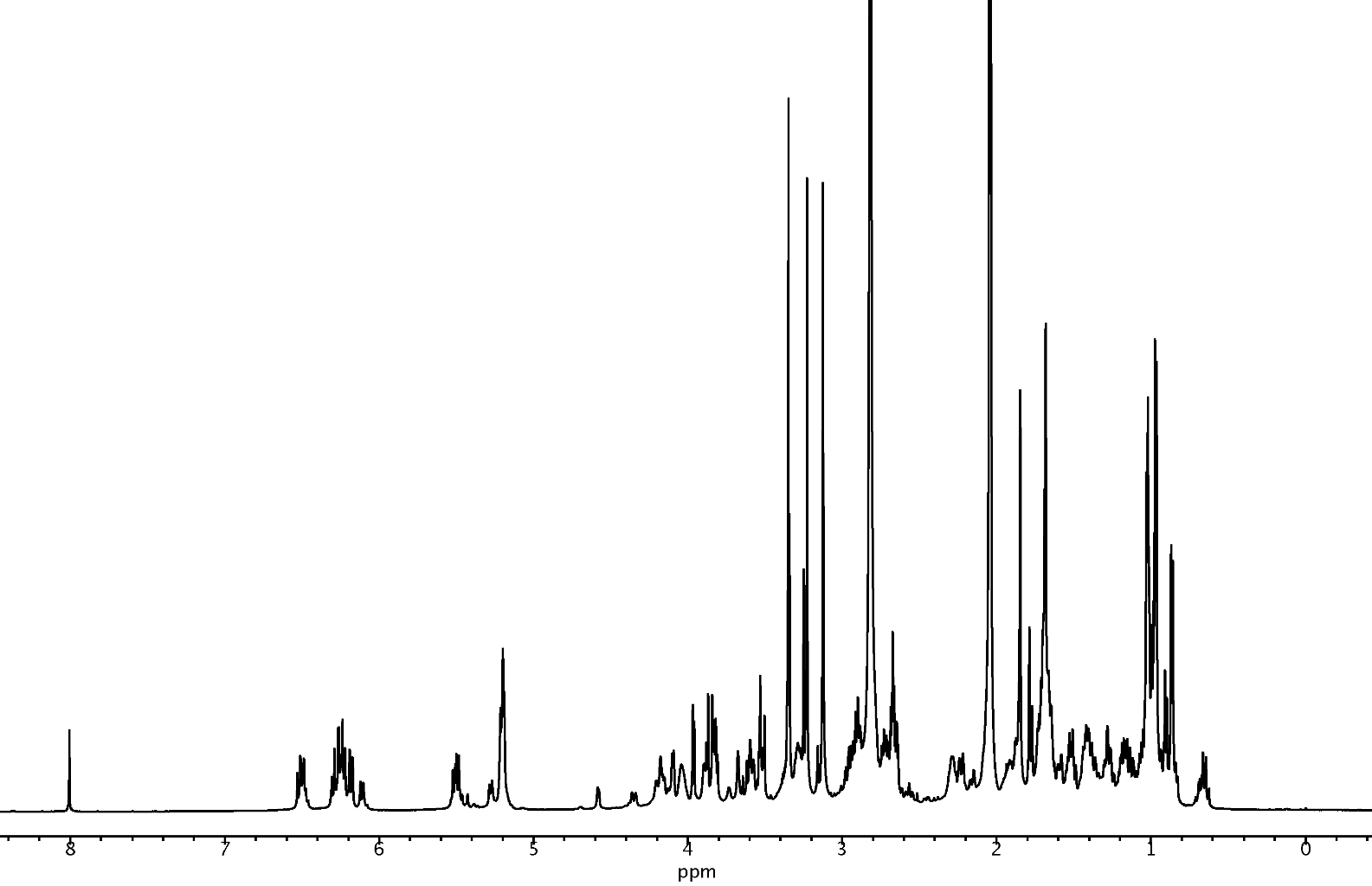
**

**Supplementary Data 2.** ^13^C NMR spectrum (Acetone-*d_6_*, 151 MHz) of compound **1**.

**^
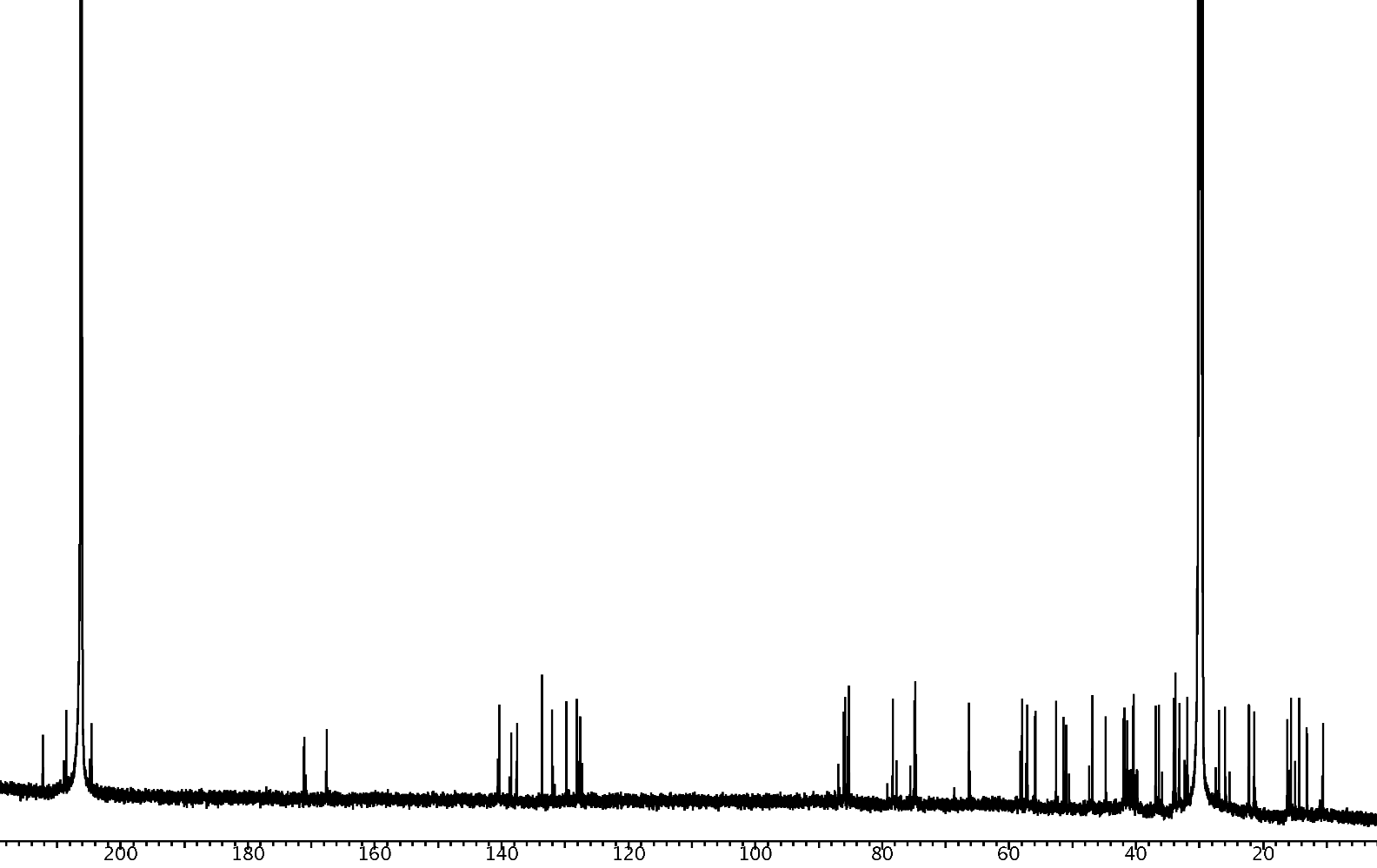
^**

**Supplementary Data 3.** Gradient DQF-COSY NMR spectrum (Acetone-*d_6_*, 600 MHz) of compound **1**.

**
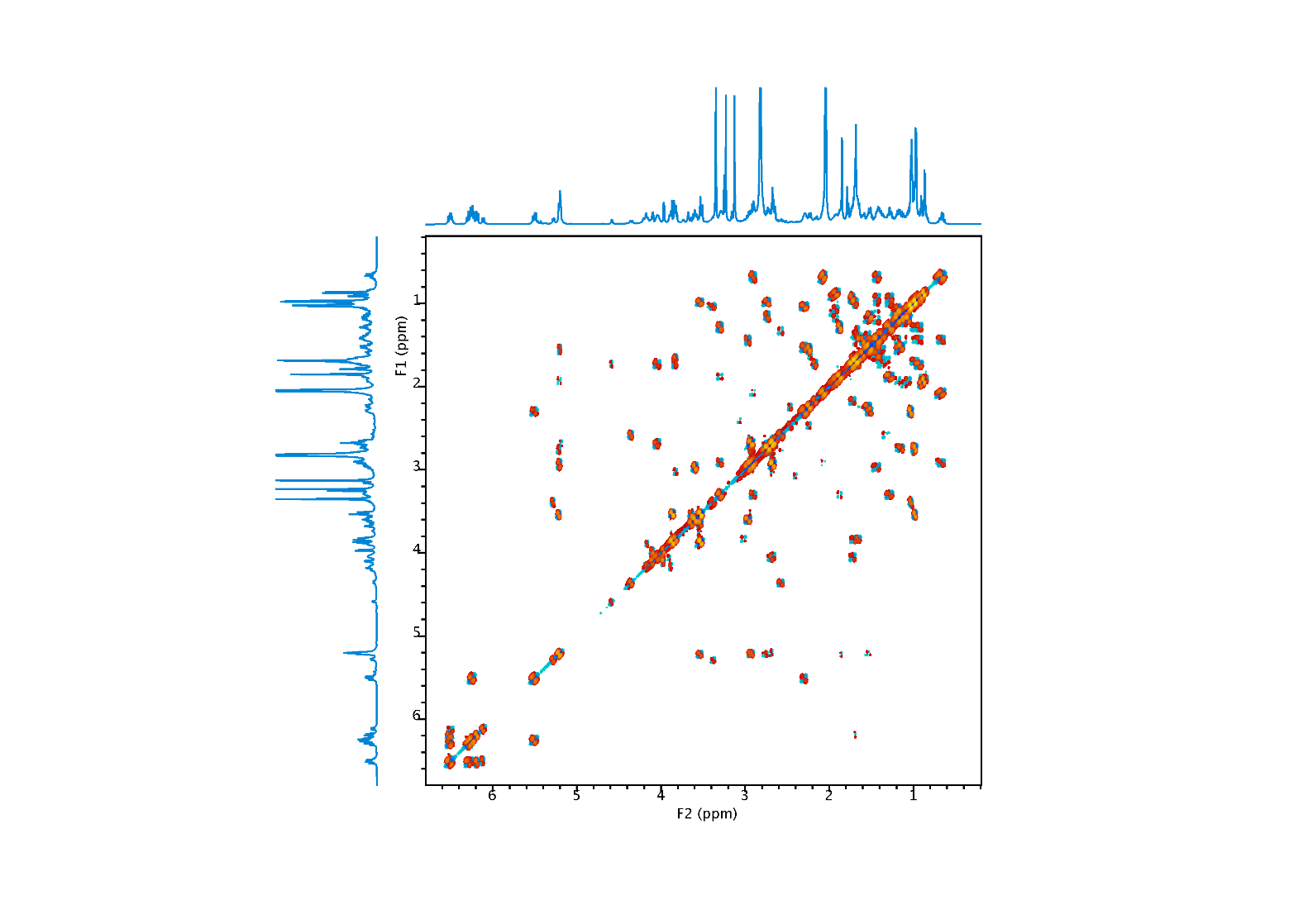
**

**Supplementary Data 4.** Gradient HSQCAD NMR spectrum (Acetone-*d_6_*, 600 MHz) of compound **1**.

**
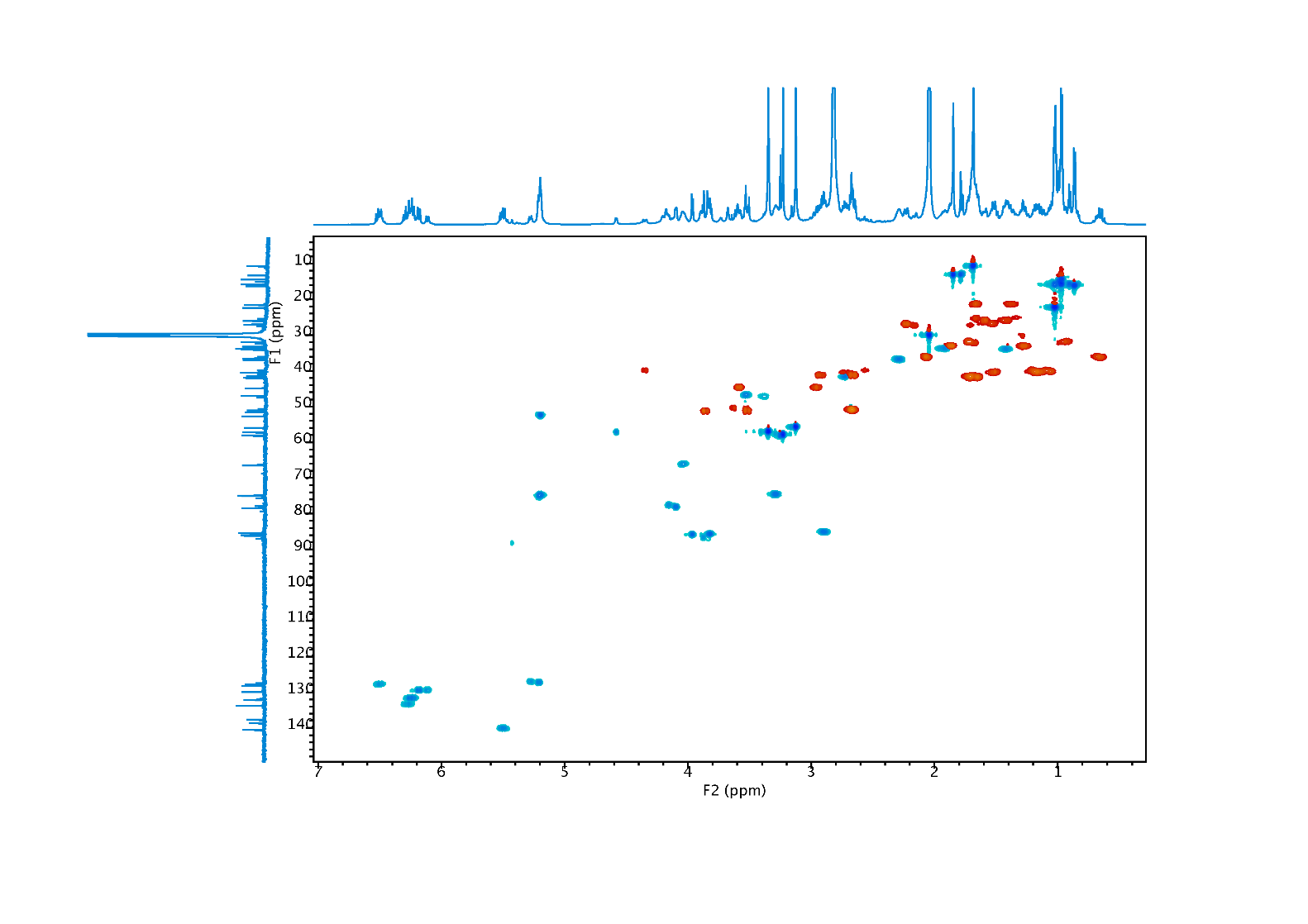
**

**Supplementary Data 5.** Gradient HMBCAD NMR spectrum (Acetone-*d_6_*, 600 MHz) of compound **1**.

**
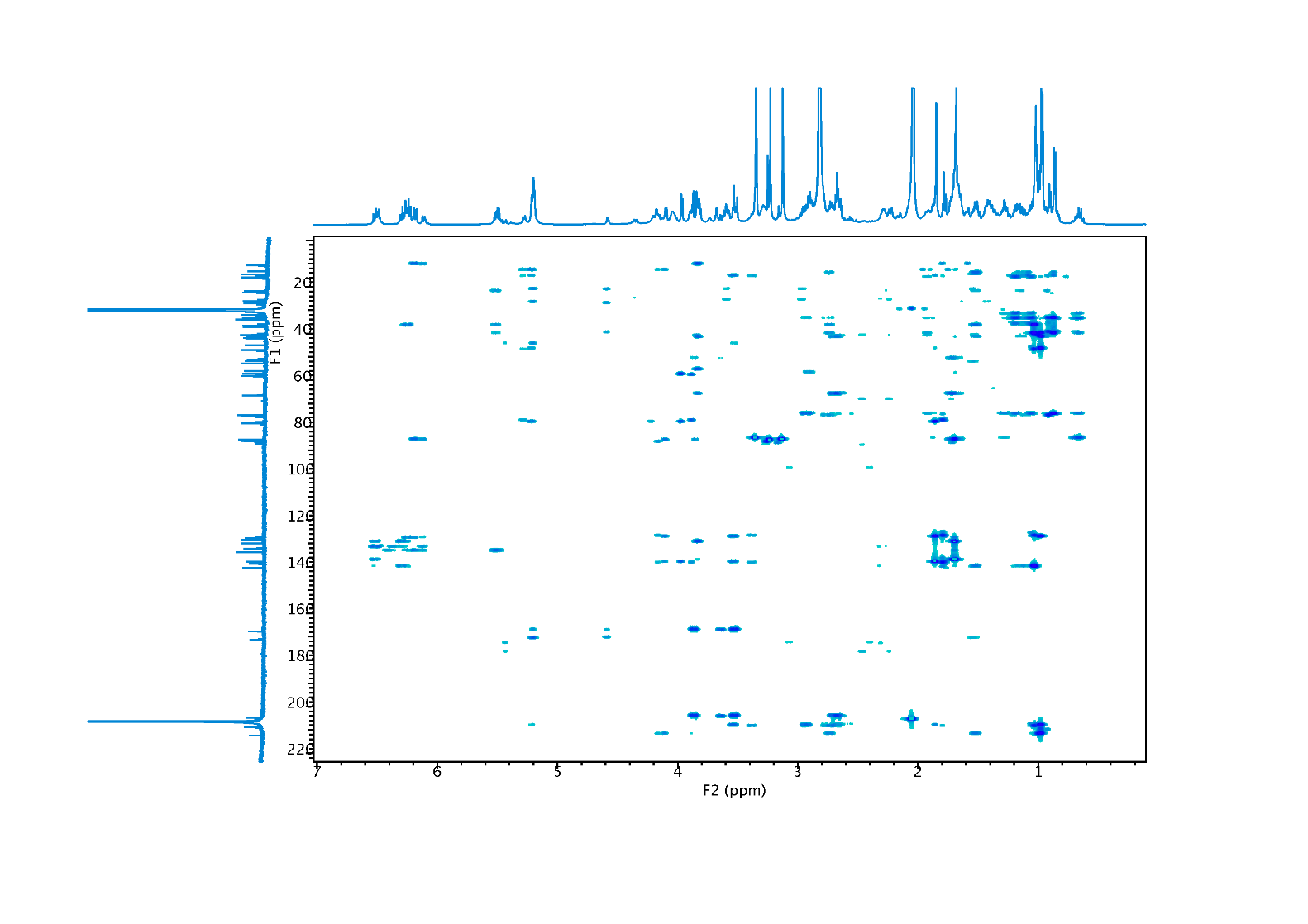
**

**Supplementary Data 6.** ^1^H NMR spectrum (Acetone-*d_6_*, 600 MHz) of compound **2**.

**
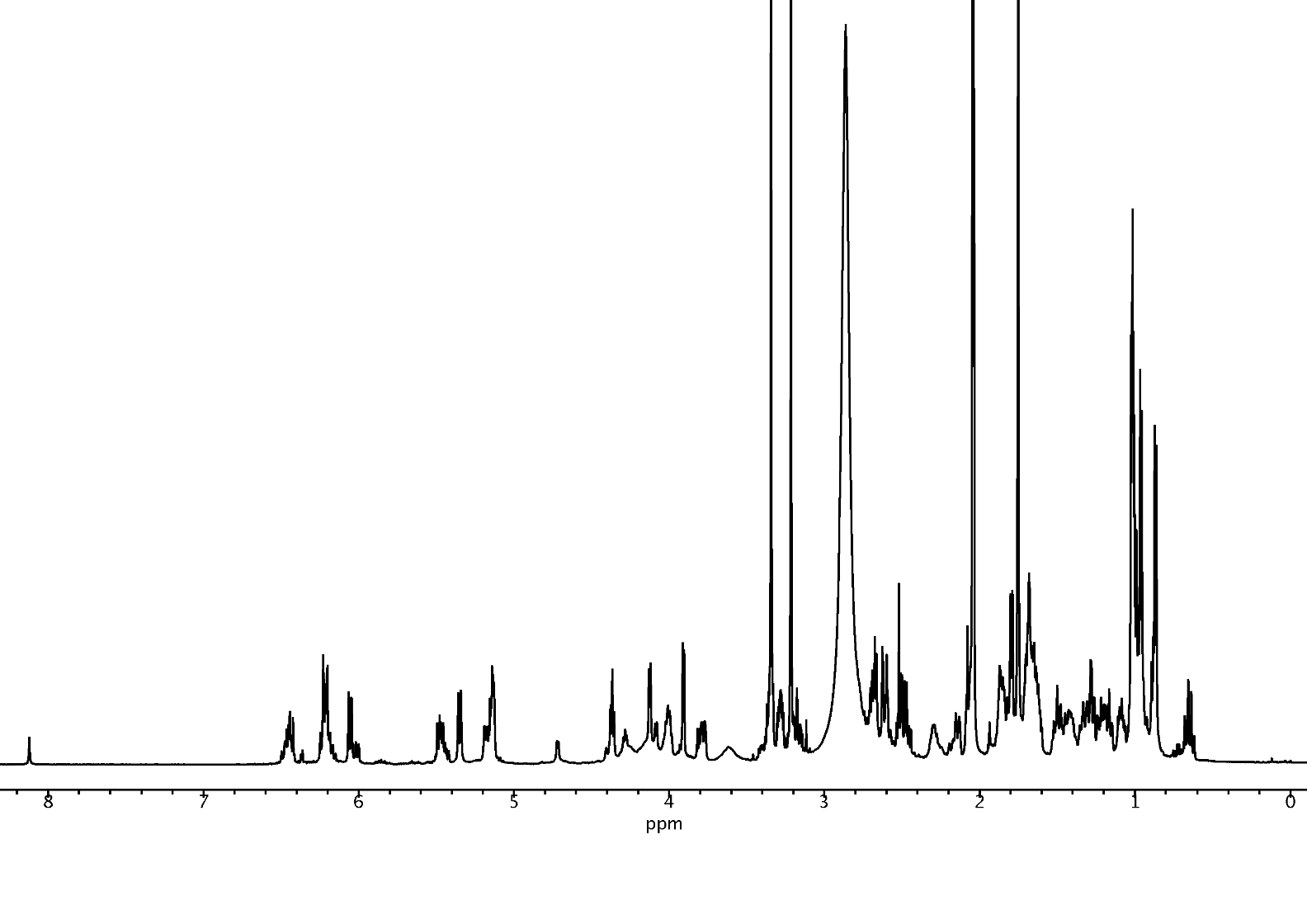
**

**Supplementary Data 7.** ^13^C NMR spectrum (Acetone-*d_6_*, 151 MHz) of compound **2**.

**
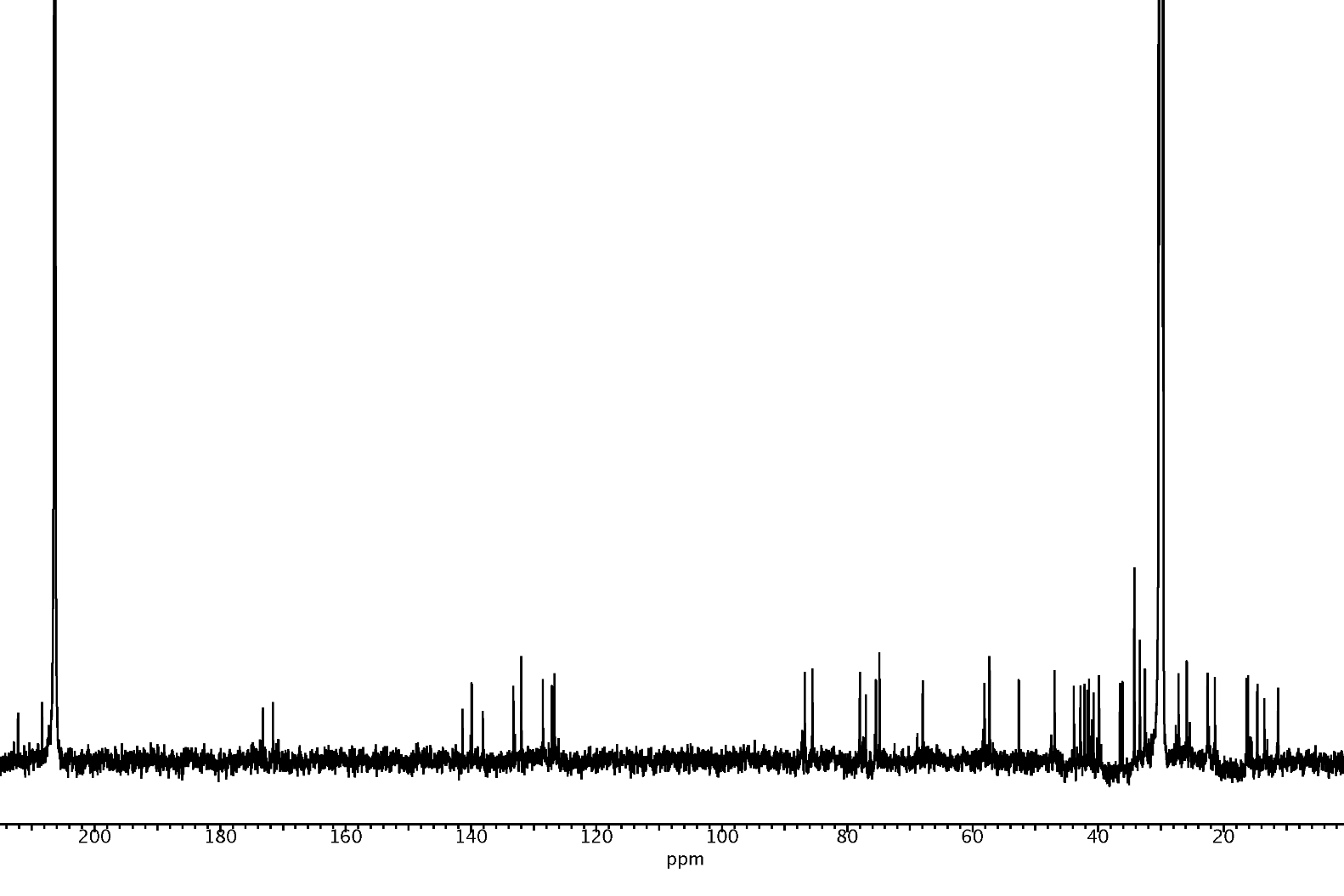
**

**Supplementary Data 8.** Gradient DQF-COSY NMR spectrum (Acetone-*d_6_*, 500 MHz) of compound **2**.

**
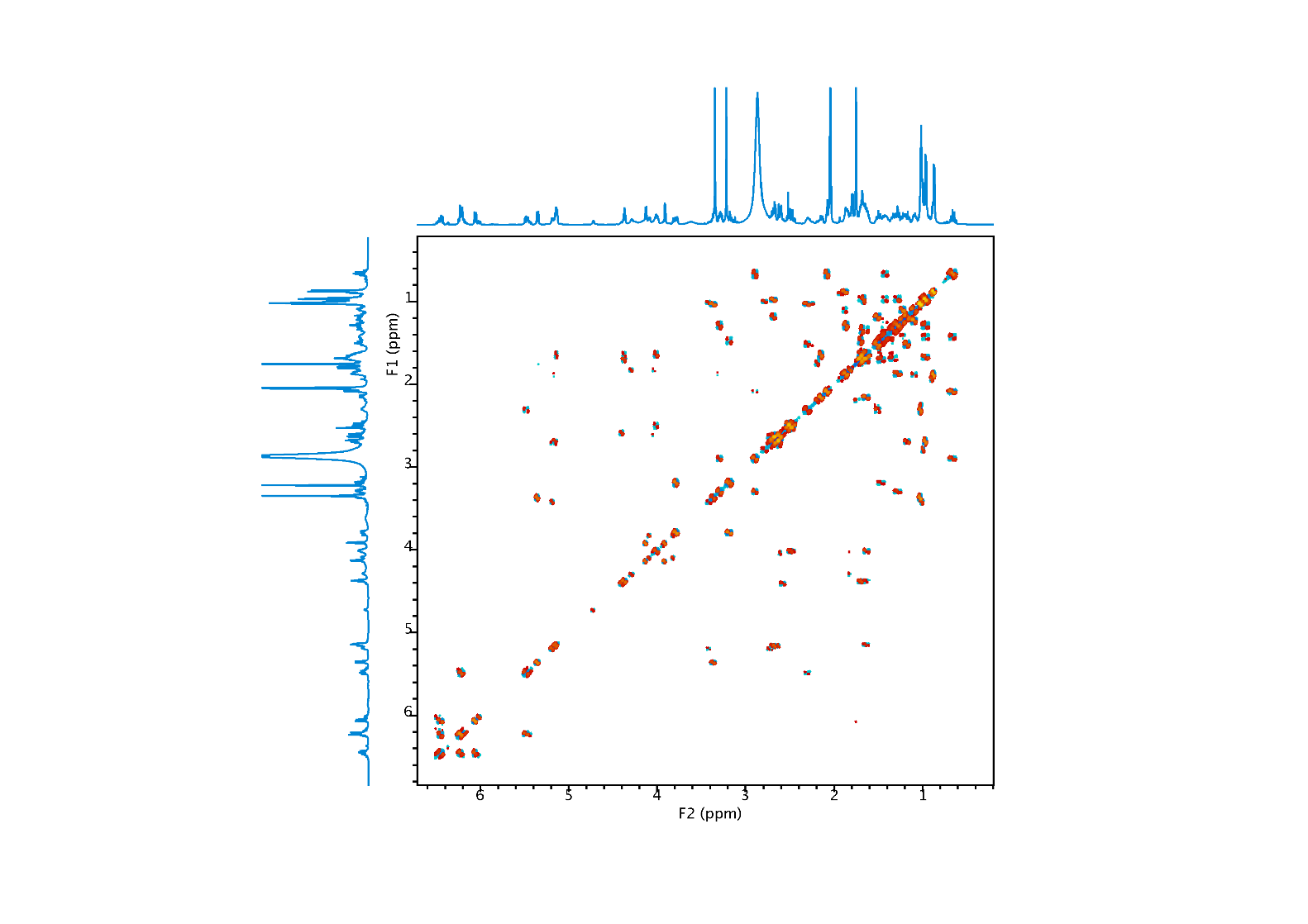
**

**Supplementary Data 9.** Gradient HSQCAD NMR spectrum (Acetone-*d_6_*, 600 MHz) of compound **2**.

**
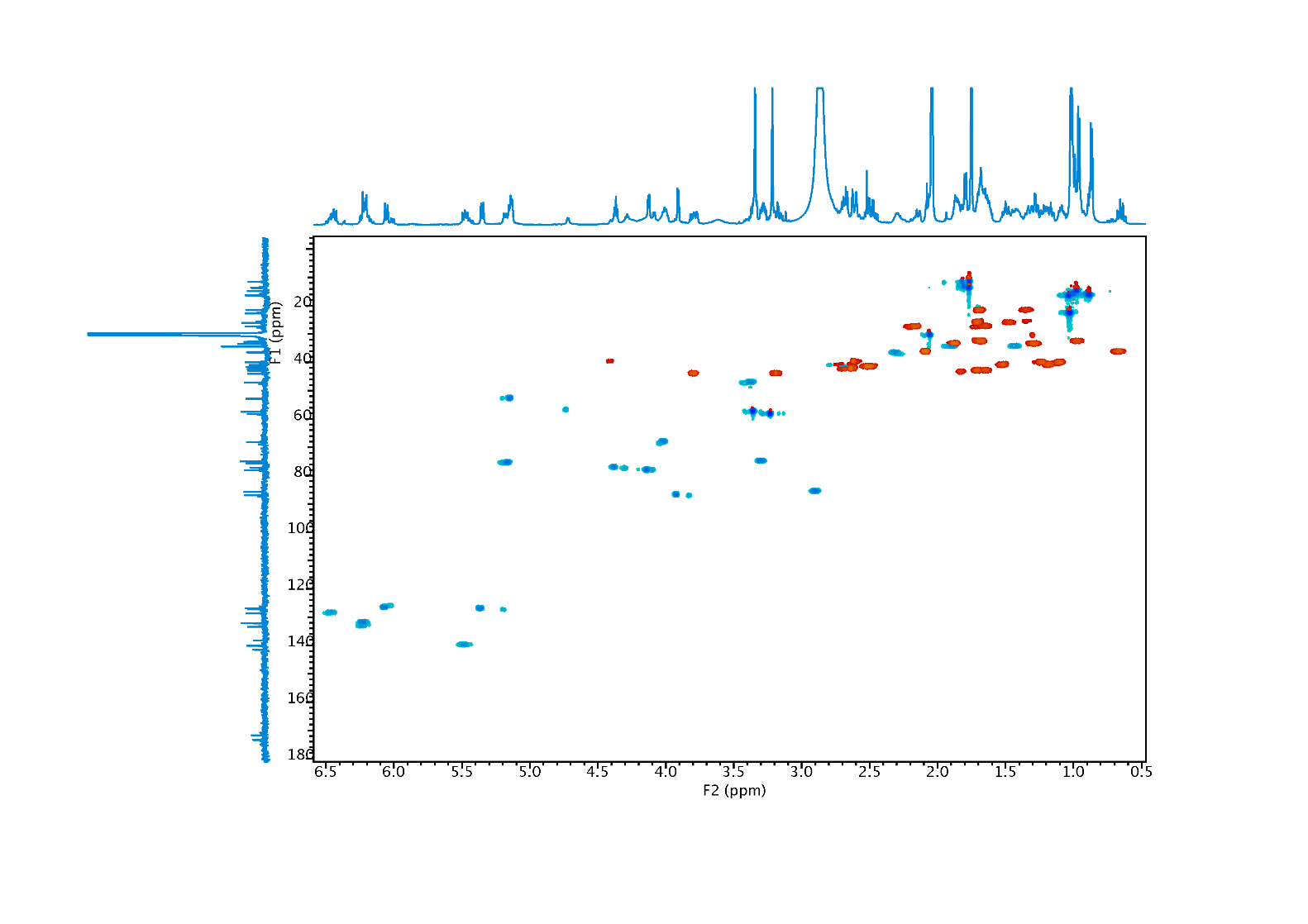
**

**Supplementary Data 10.** Gradient HMBCAD NMR spectrum (Acetone-*d_6_*, 600 MHz) of compound **2**.

**
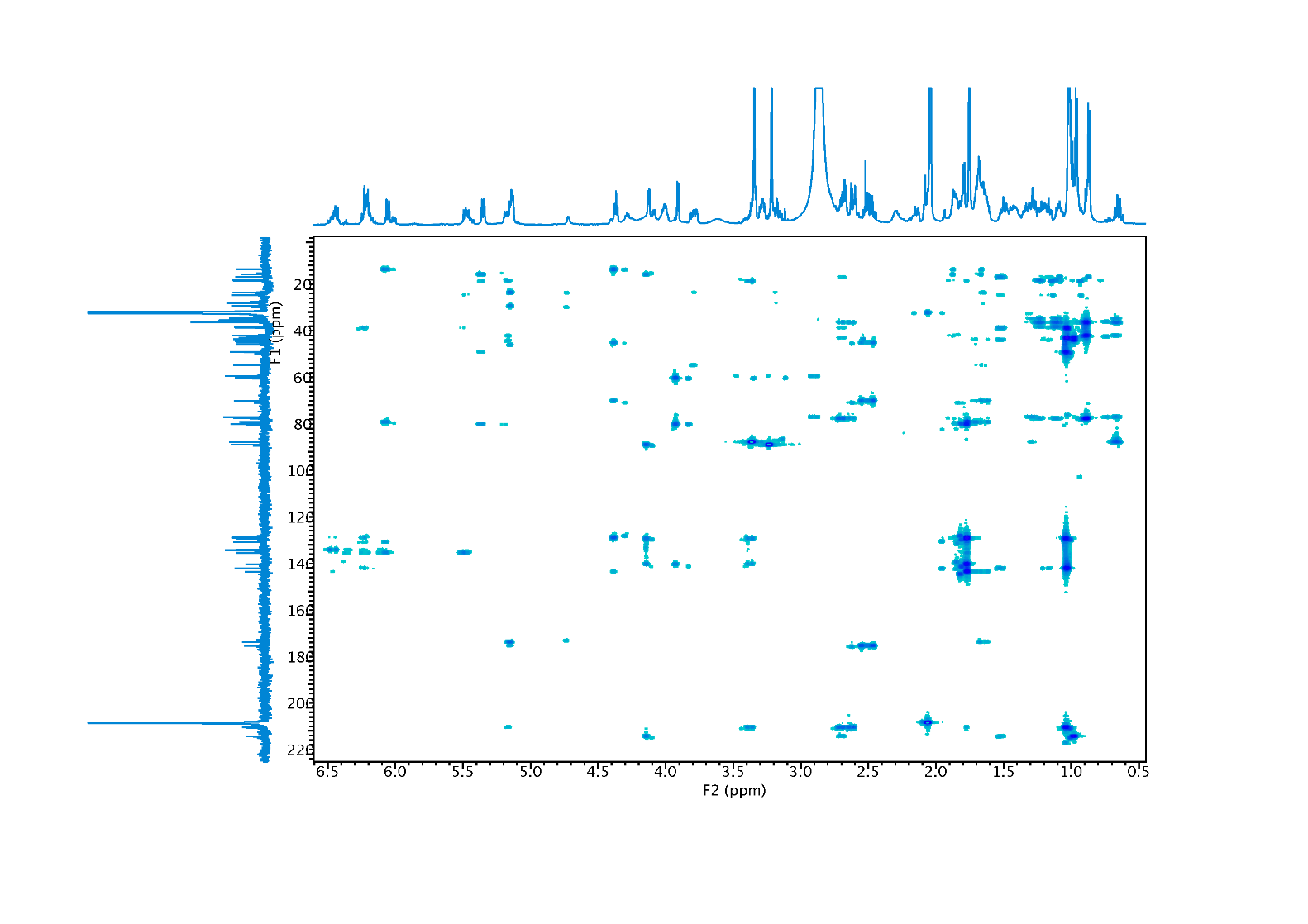
**

**Supplementary Data 11.** ^1^H NMR spectrum (Acetone-*d_6_*, 500 MHz) of compound **3**.

**^
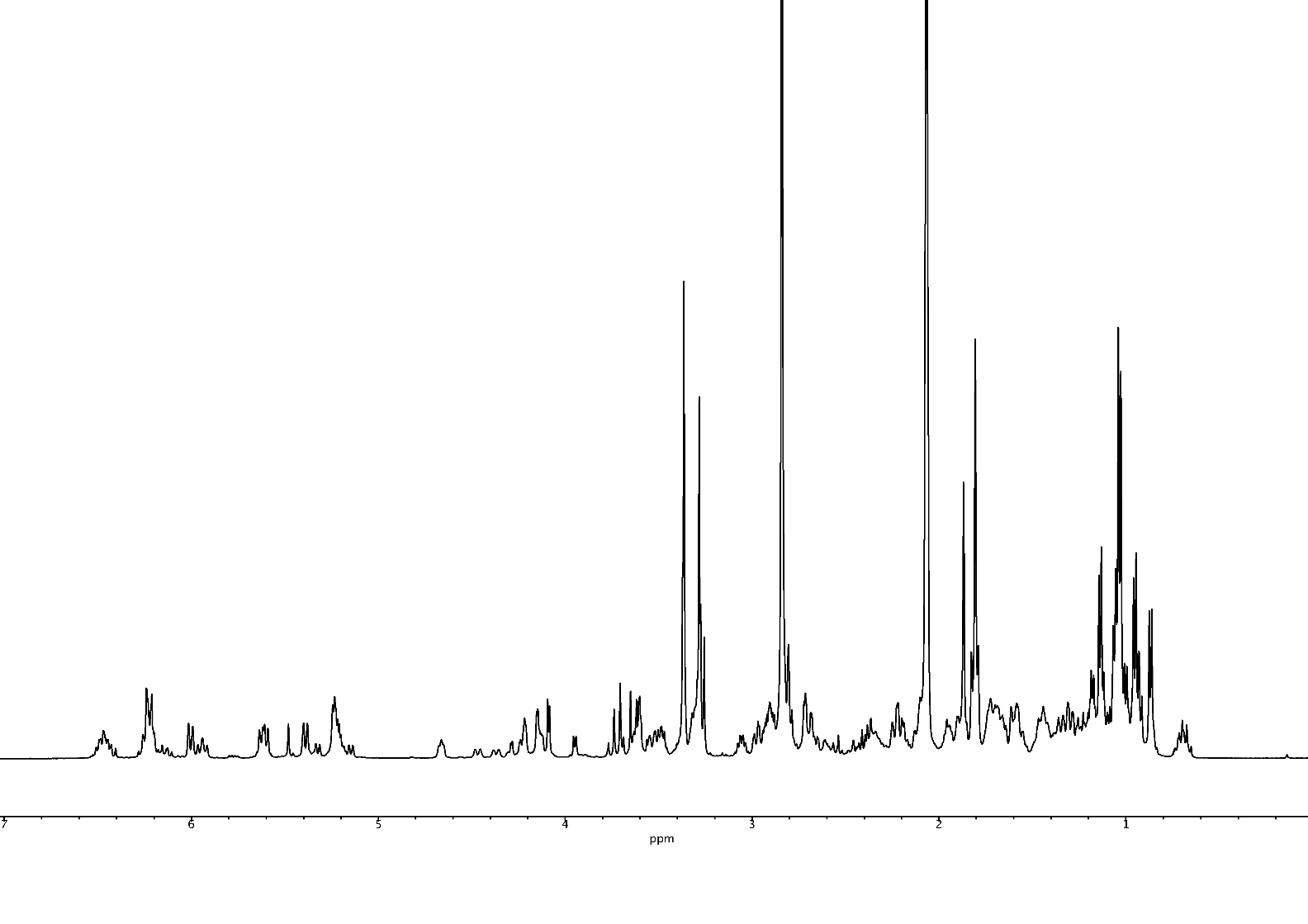
^**

**Supplementary Data 12.** ^13^C NMR spectrum (Acetone-*d_6_*, 125 MHz) of compound **3**.

**
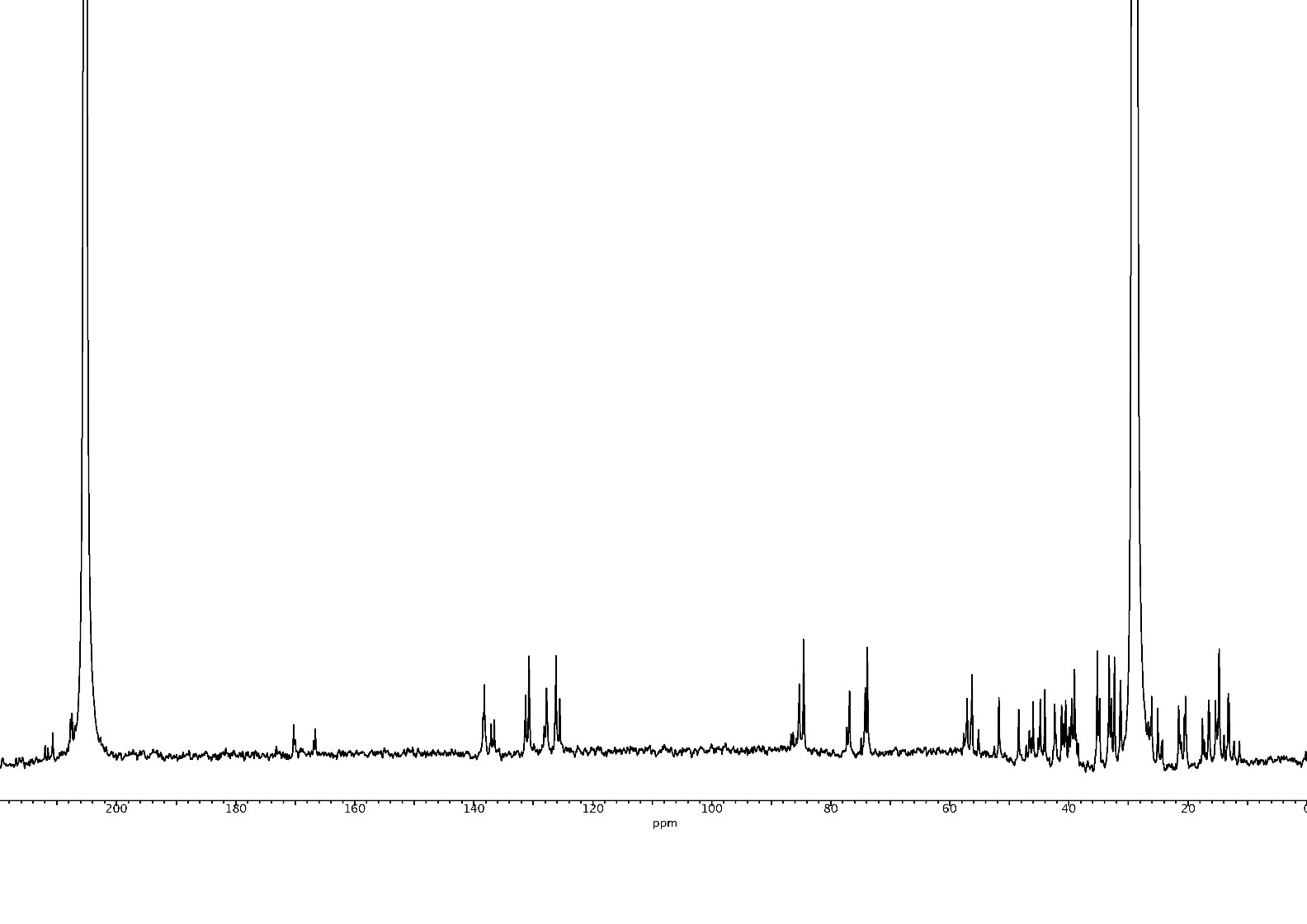
**

**Supplementary Data 13.** Gradient DQF-COSY NMR spectrum (Acetone-*d_6_*, 500 MHz) of compound **3**.

**^
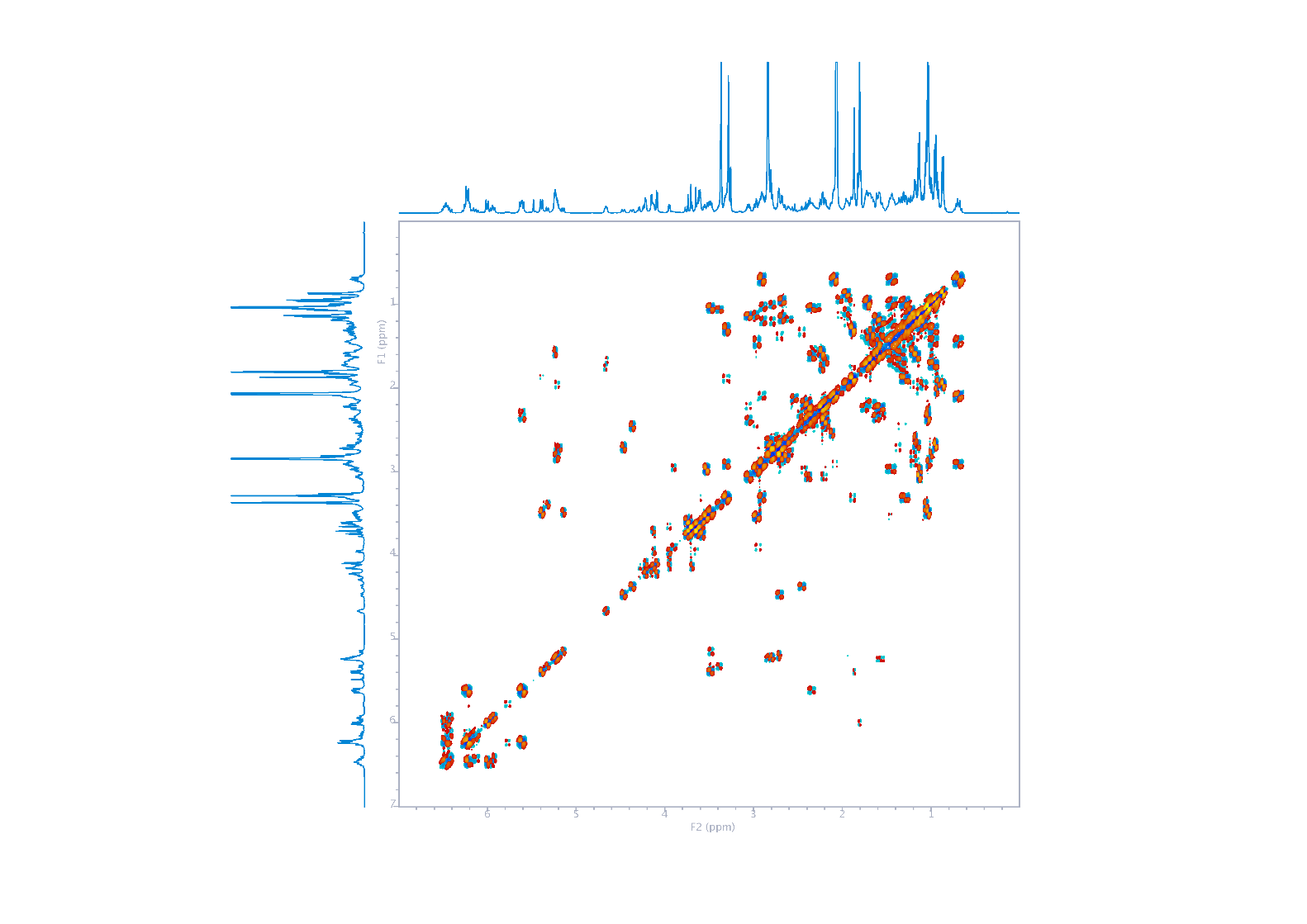
^**

**Supplementary Data 14.** Gradient HSQCAD NMR spectrum (Acetone-*d_6_*, 500 MHz) of compound **3**.

**
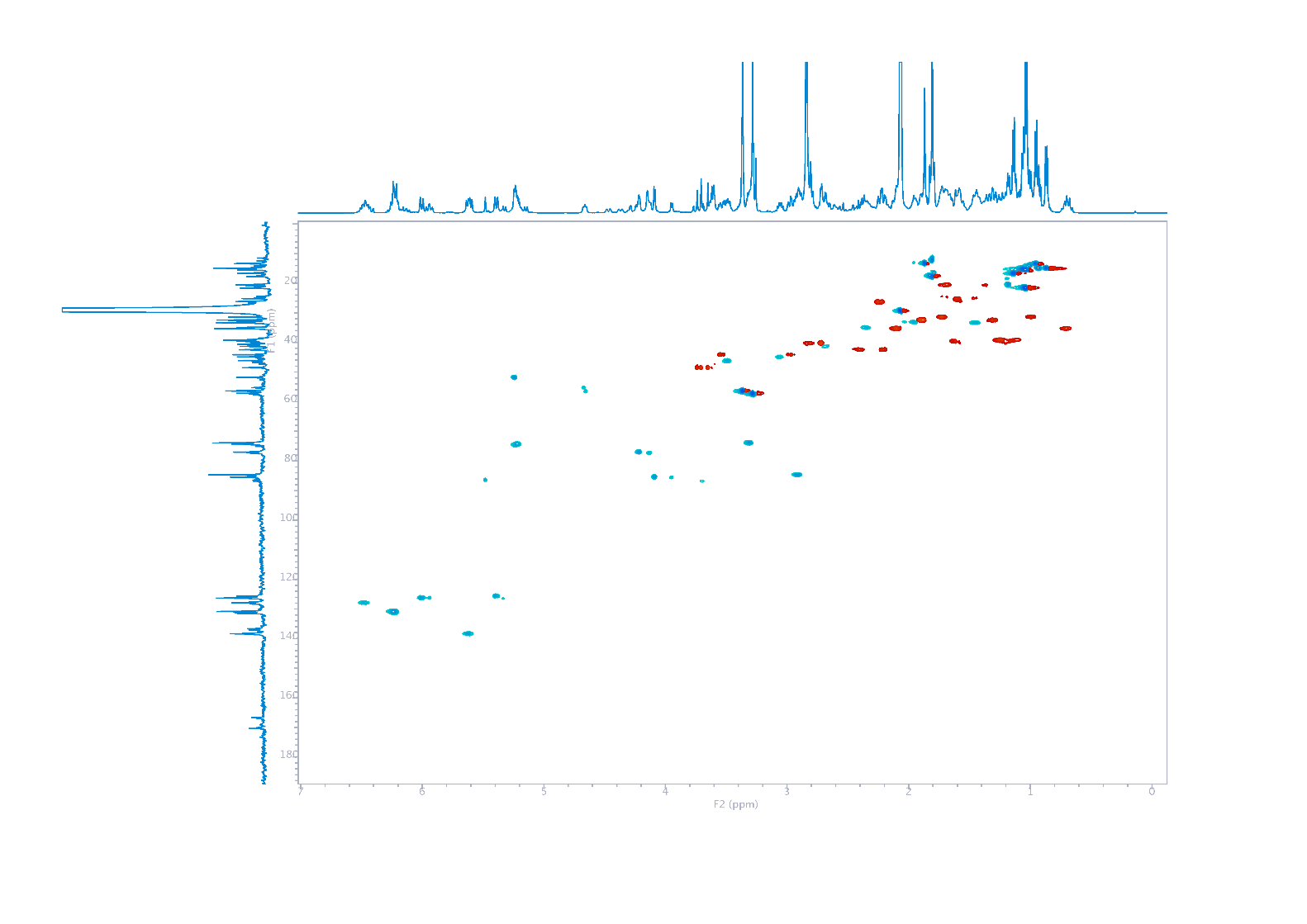
**

**Supplementary Data 15.** Gradient HMBCAD NMR spectrum (Acetone-*d_6_*, 500 MHz) of compound **3**.

**
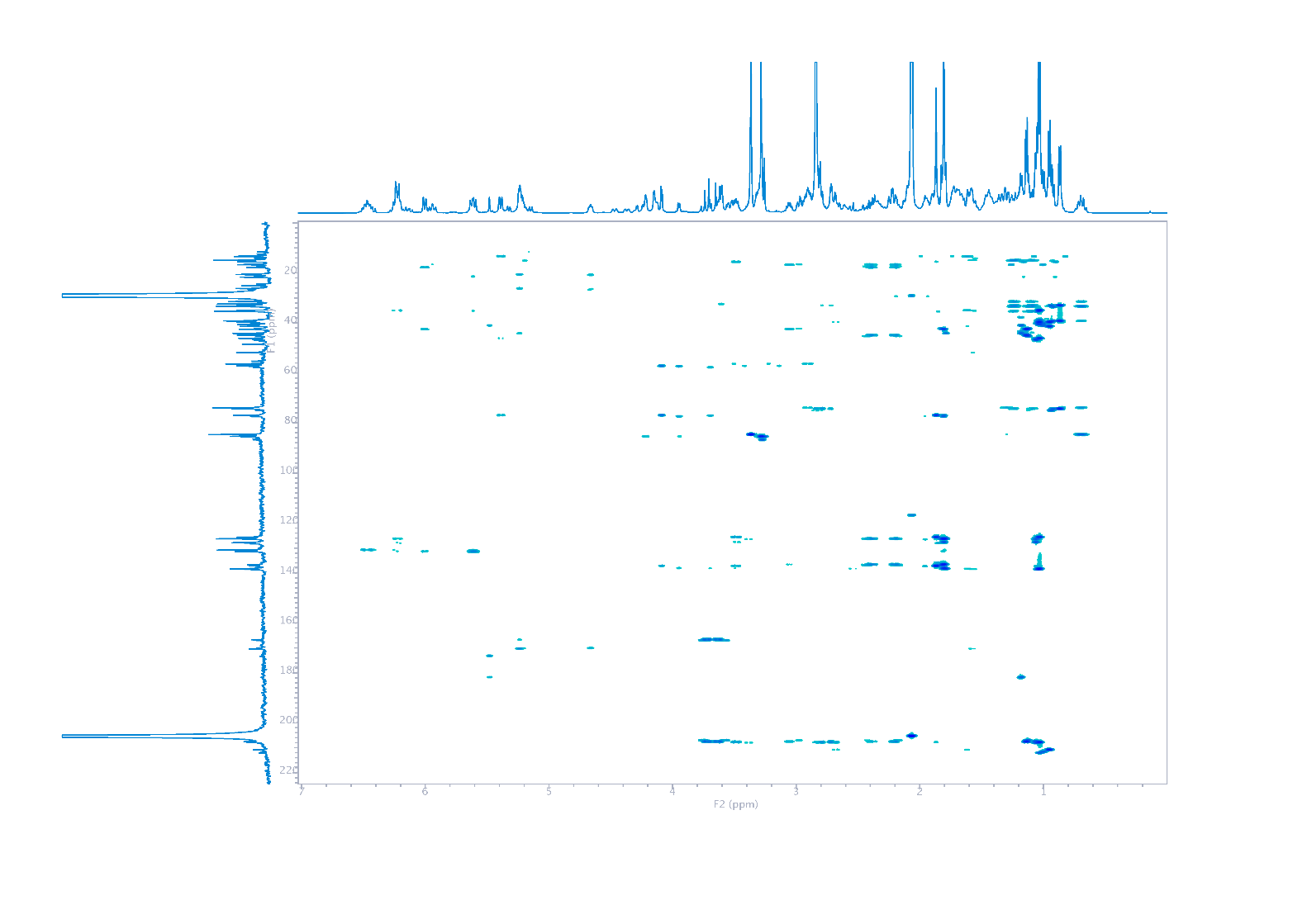
**

**Supplementary Data 16.** ^1^H NMR spectrum (Acetone-*d_6_*, 500 MHz) of compound **4**.

**
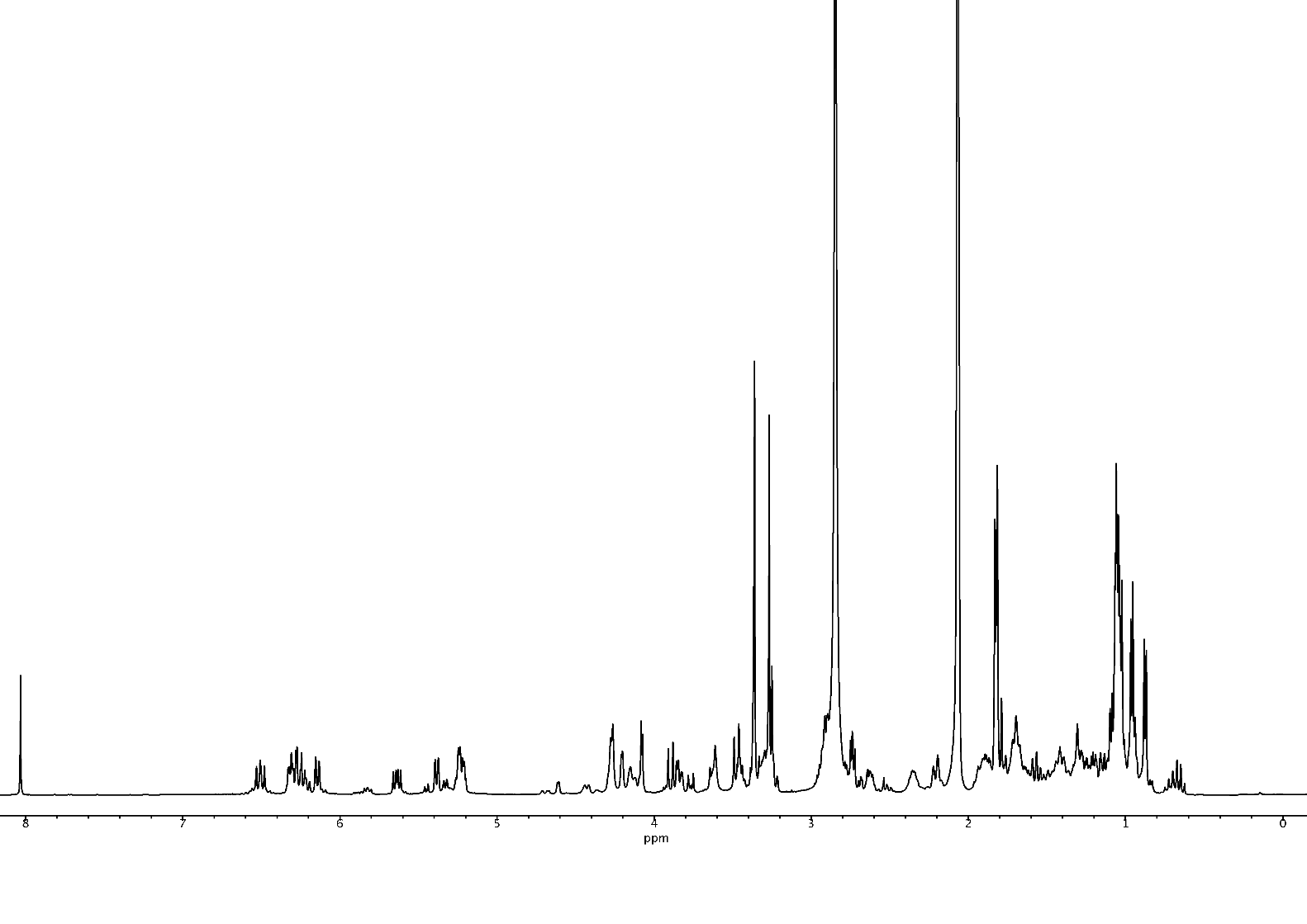
**

**Supplementary Data 17.** ^13^C NMR spectrum (Acetone-*d_6_*, 151 MHz) of compound **4**.

**
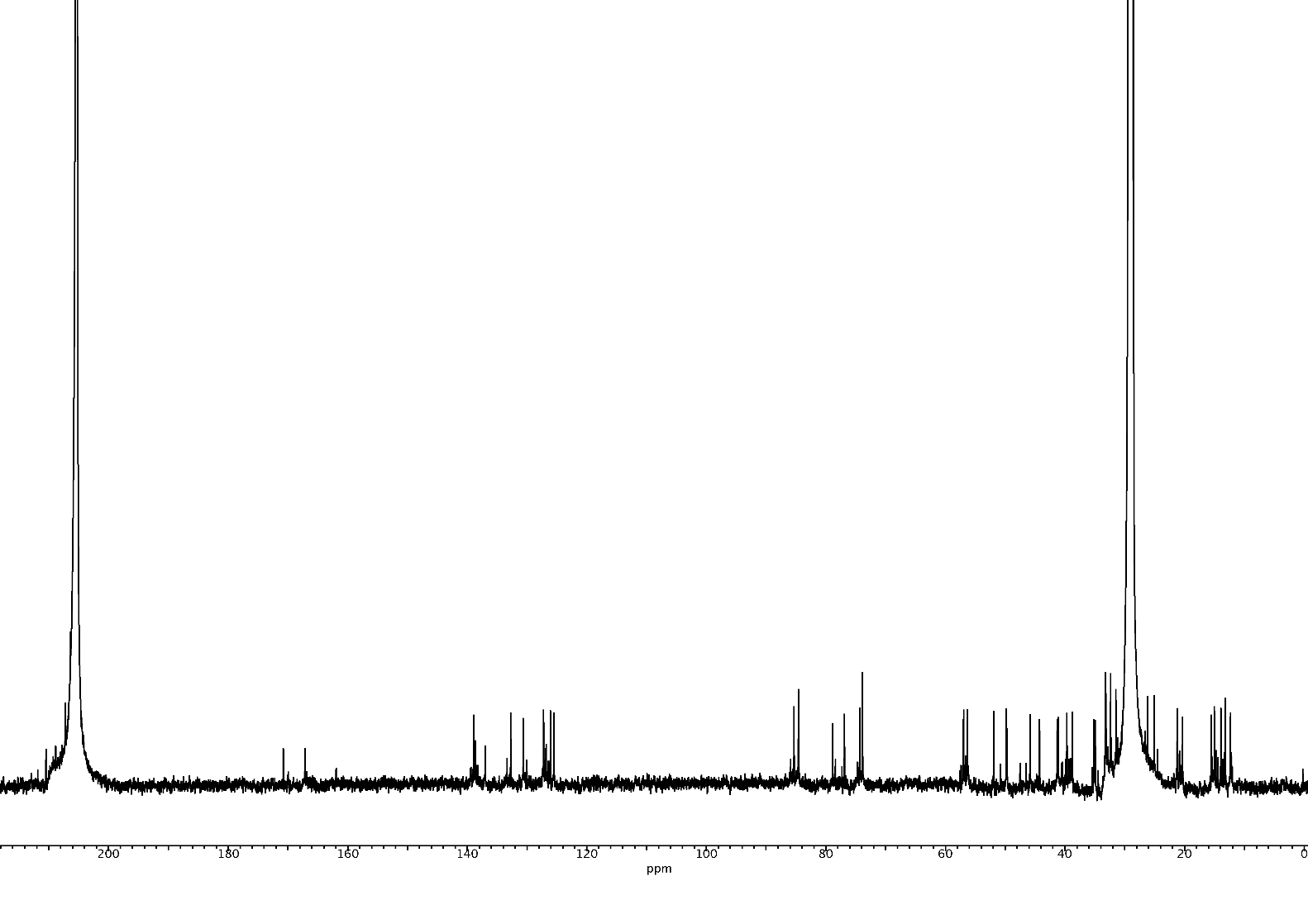
**

**Supplementary Data 18.** Gradient DQF-COSY NMR spectrum (Acetone-*d_6_*, 500 MHz) of compound **4**.

**
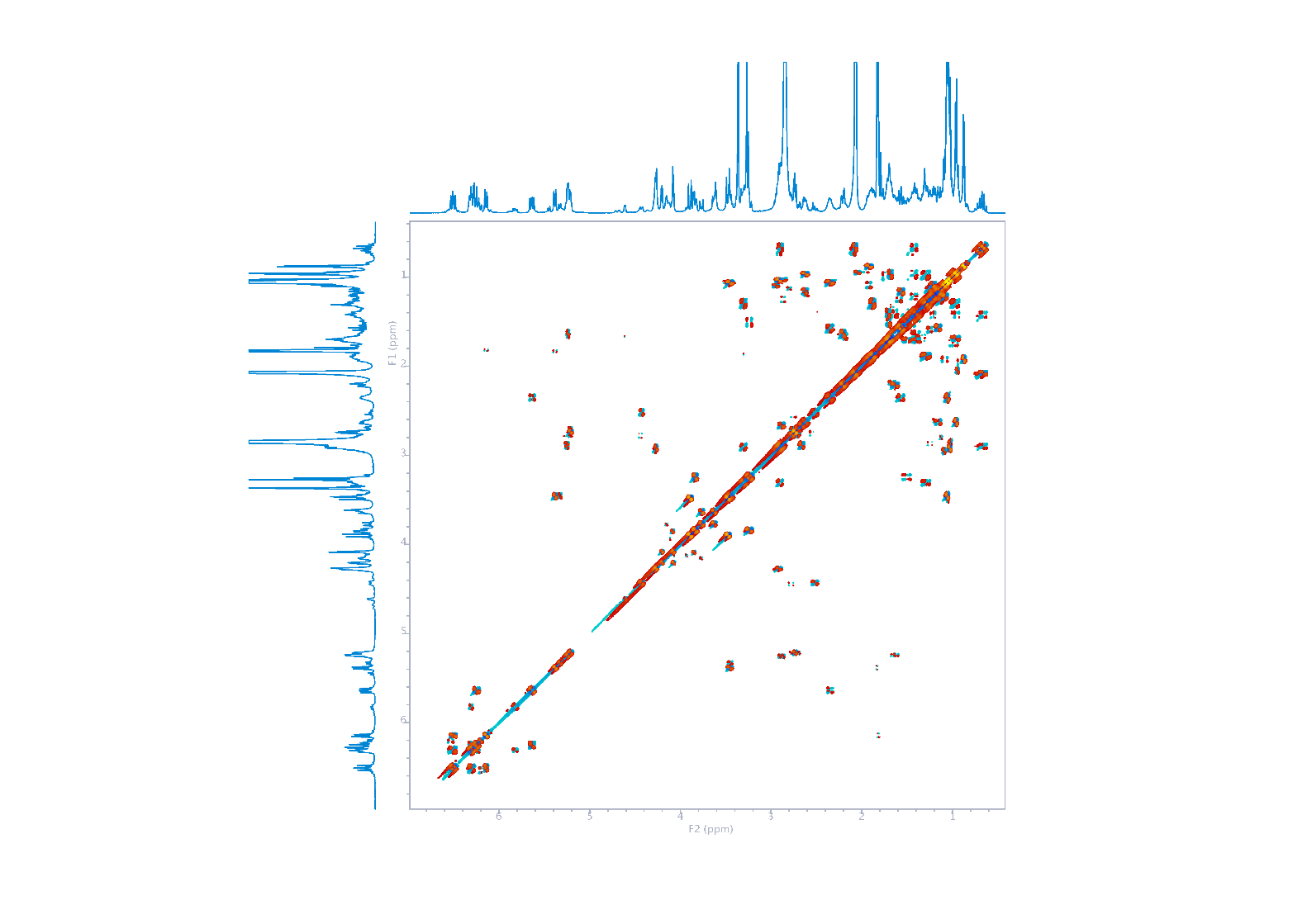
**

**Supplementary Data 19.** Gradient HSQCAD NMR spectrum (Acetone-*d_6_*, 500 MHz) of compound **4**.

**
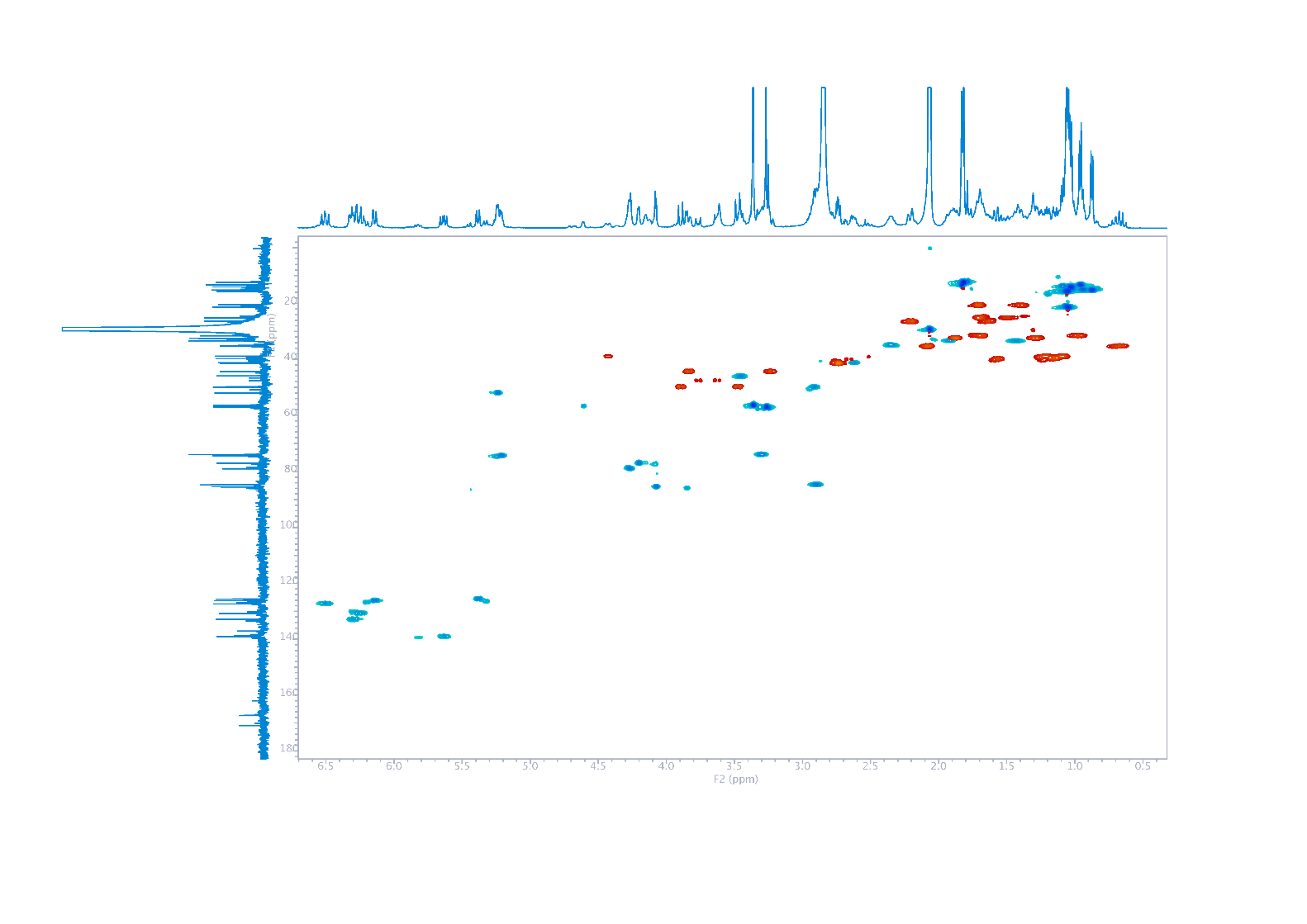
**

**Supplementary Data 20.** Gradient HMBCAD NMR spectrum (Acetone-*d_6_*, 500 MHz) of compound **4**.

**
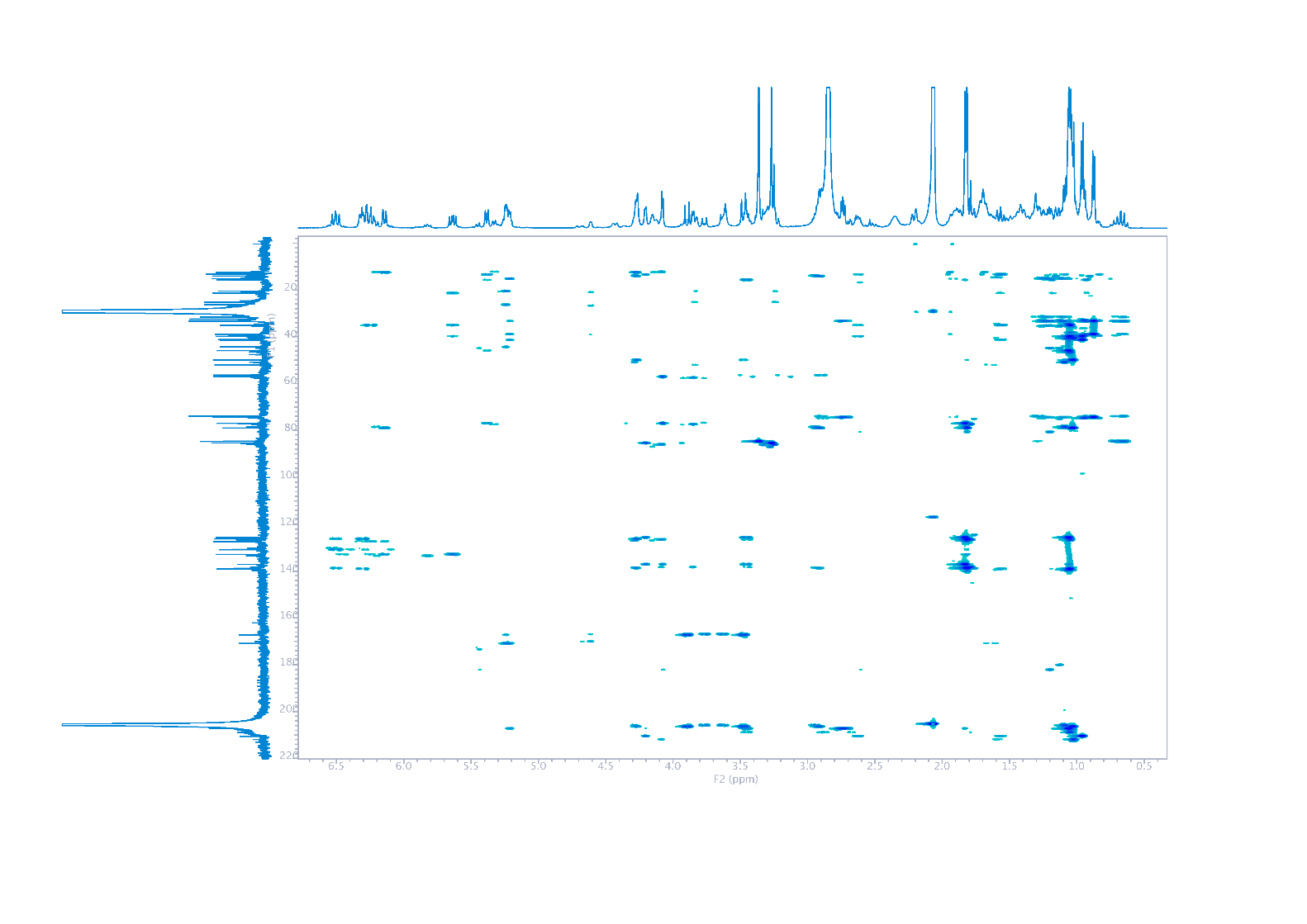
**
